# The *lethal giant larvae *tumour suppressor mutation requires dMyc oncoprotein to promote clonal malignancy

**DOI:** 10.1186/1741-7007-8-33

**Published:** 2010-04-07

**Authors:** Francesca Froldi, Marcello Ziosi, Flavio Garoia, Andrea Pession, Nicola A Grzeschik, Paola Bellosta, Dennis Strand, Helena E Richardson, Annalisa Pession, Daniela Grifoni

**Affiliations:** 1 Alma Mater Studiorum, Dipartimento di Patologia Sperimentale, Via S. Giacomo 14, 40126 Bologna, Italy; 2 Alma Mater Studiorum, Dipartimento di Biologia Evoluzionistica Sperimentale, Via Selmi 3, 40126 Bologna, Italy; 3 NGB Genetics s.r.l., University of Ferrara, Via Borsari 46, 44100 Ferrara, Italy; 4 Alma Mater Studiorum, Dipartimento di Ginecologia, Ostetricia e Pediatria, Via Massarenti 9, 40138 Bologna, Italy; 5 Peter MacCallum Cancer Centre, Research Division, 7 St Andrew's Place, East Melbourne, Victoria 3002, Australia; 6 City College of the City University of New York, Department of Biology, Convent Ave at 138th, New York, NY 10031, USA; 7 Johannes Gutenberg University, First Department of Internal Medicine, 63 Obere Zahlbacherstr, 55131 Mainz, Germany; 8 Department of Anatomy and Cell Biology and Department of Biochemistry and Molecular Biology, University of Melbourne, Parkville, Victoria 3052, Australia

## Abstract

**Background:**

Neoplastic overgrowth depends on the cooperation of several mutations ultimately leading to major rearrangements in cellular behaviour. Precancerous cells are often removed by cell death from normal tissues in the early steps of the tumourigenic process, but the molecules responsible for such a fundamental safeguard process remain in part elusive. With the aim to investigate the molecular crosstalk occurring between precancerous and normal cells *in vivo*, we took advantage of the clonal analysis methods that are available in *Drosophila *for studying the phenotypes due to *lethal giant larvae *(*lgl*) neoplastic mutation induced in different backgrounds and tissues.

**Results:**

We observed that *lgl *mutant cells growing in wild-type imaginal wing discs show poor viability and are eliminated by Jun N-terminal Kinase (JNK)-dependent cell death. Furthermore, they express very low levels of dMyc oncoprotein compared with those found in the surrounding normal tissue. Evidence that this is a cause of *lgl *mutant cells elimination was obtained by increasing dMyc levels in *lgl *mutant clones: their overgrowth potential was indeed re-established, with mutant cells overwhelming the neighbouring tissue and forming tumourous masses displaying several cancer hallmarks. Moreover, when *lgl *mutant clones were induced in backgrounds of slow-dividing cells, they upregulated dMyc, lost apical-basal cell polarity and were able to overgrow. Those phenotypes were abolished by reducing dMyc levels in the mutant clones, thereby confirming its key role in *lgl*-induced tumourigenesis. Furthermore, we show that the *eiger*-dependent Intrinsic Tumour Suppressor pathway plays only a minor role in eliminating *lgl *mutant cells in the wing pouch; *lgl*^-/- ^clonal death in this region is instead driven mainly by dMyc-induced Cell Competition.

**Conclusions:**

Our results provide the first evidence that dMyc oncoprotein is required in *lgl *tumour suppressor mutant tissue to promote invasive overgrowth in larval and adult epithelial tissues. Moreover, we show that dMyc abundance inside *versus *outside the mutant clones plays a key role in driving neoplastic overgrowth.

## Background

In *Drosophila*, the tumour suppressor gene *lethal giant larvae *(*lgl*) functions together with *scribble *(*scrib*) and *discs large *(*dlg*) to link apical-basal cell polarity regulation to cell proliferation control in epithelial tissues [1,2]. Lgl, Dlg and Scrib are scaffold proteins associated with septate junctions at the lateral membrane, where they function in establishing basolateral domain identity by antagonising the activity of two other complexes: the Bazooka (Baz/Par3)/Par6/atypical Protein Kinase C (aPKC) and the Crumbs (Crb)/Stardust (Sdt)/PATJ complexes, which define the apical membrane domain [3]. Alterations to this process compromise apical-basal and cytoskeletal structure and eventually disrupt epithelial integrity [2]. When the whole individual is mutant for *lgl*, loss of cell structure and neoplastic overgrowth indeed occur, despite a low proliferation rate, in larval imaginal epithelia, monolayered diploid organs which give rise to the adult appendages. These epithelia grow as highly disorganised, multistratified masses in which cell differentiation does not occur and the animal dies after a prolonged larval stage [1,2]. Furthermore, when these tumourous tissues are transplanted into wild-type recipients, they overgrow and cells migrate to distant sites where they are able to form secondary tumours [4], thus showing some of the properties of mammalian metastatic cancers. The function of *lgl *as a tumour suppressor is evolutionarily conserved, since the human orthologue, *Hugl-1*, can rescue *Drosophila lgl *mutants, and downregulation of its activity in human cells results in cell polarity and proliferation defects and is associated with cancer [5,6]. Despite the malignant behaviour (increased tumour growth and survival and invasive properties) of *lgl *mutant tissues, adult wings from *lgl*^+/- ^individuals in which *lgl*^-/- ^clones had been generated at the larval stage by X-rays did not display tumourous growth [7], raising the possibility that *lgl *mutant cells could be eliminated through a mechanism induced by the surrounding tissue.

There are two mechanisms described in the literature that may act to prevent *lgl*^-/- ^clones from developing into tumours. First, it has been recently shown that the growth of polarity-deficient *scrib *or *dlg *mutant clones is counteracted by the so-called *Intrinsic Tumour Suppression *(ITS) mechanism, which functions by triggering in the mutant cells both endocytic uptake and endosomal activation of the Eiger protein (homologue of the mammalian Tumour Necrosis Factor), which in turn leads to activation of pro-apoptotic JNK signaling [8,9]. However, when normal tissue surrounding the mutant clones is removed, mutant cells are no longer eliminated and grow into tumours [8]. ITS can be thus classified as a non-autonomous process, in which an as yet unknown signal from the normal tissue acts to induce the mutant cells to undergo apoptosis. Second, it has long been known that relatively slow-growing cells are eliminated from developing tissues by a phenomenon termed *Cell Competition *(CC), where slow-dividing cells are killed and replaced by the surrounding faster-growing populations, eventually giving rise to an organ of proper size [10]. CC was first described by analysing *Minute *(*M*) mutations in *Drosophila *[11], a group of dominant, homozygous-lethal mutations of various ribosomal genes. *M/*+ cells are viable in a homotypic context (that is, all the cells composing the tissue are of the same genotype) though showing a reduced rate of cell proliferation; however, when juxtaposed to wild-type cells, they are eliminated by apoptosis and the wild-type cells overtake the *M/*+ cells so that the organ eventually reaches normal size. It has been recently speculated that these competitive interactions could have evolved as a mechanism ensuring that viable but unfit, damaged or potentially harmful cells do not accumulate within a growing tissue, and this could be the case in normal development as well as in tumourigenesis [12,13]. During recent years it has become evident that different levels of dMyc protein in adjacent cell populations can trigger competitive interactions; cells with higher dMyc levels are able to out-compete those showing lower levels by inducing apoptotic death [14,15]. Both *Minute *and dMyc-induced CC are assumed to depend on a local difference in ribosome biogenesis or function that affects cell growth and proliferation [13]. Clonal alterations in dMyc expression are indeed detrimental due to its key role in cell growth, proliferation and biosynthesis [16] and upregulation of this protein is associated with almost all human cancers [17], among which carcinomas show in addition alterations in apical-basal cell polarity [18,19]. *lgl *mutant cells have both abnormal polarity and a reduced proliferation rate, so both ITS and CC may be involved in their elimination. In *Drosophila *epithelia, two main pathways are known to trigger apoptosis upon polarity alteration: the JNK signalling and the Hid/dIAP1 pathways [20]. JNK signalling has been clearly associated to ITS, while its involvement in dMyc-induced CC is still controversial [14,15].

In order to identify possible molecules and mechanisms associated with the phenotype of *lgl *mutant cells, we induced *lgl*^-/- ^clones in diverse backgrounds and tissues. We show that *lgl*^-/- ^clonal phenotype strictly depends on dMyc oncoprotein levels: when *lgl*^-/- ^cells have lower levels of dMyc with respect to the surrounding tissue, their clonal growth is restricted by JNK-mediated apoptosis, whereas when *lgl*^-/- ^cells have high levels of dMyc, mutant clones survive and proliferate to form invasive tumours.

## Results and Discussion

### *lgl *mutant clones die by caspase-dependent apoptosis when induced in an *lgl*^+/- ^background

**Figure 1:**
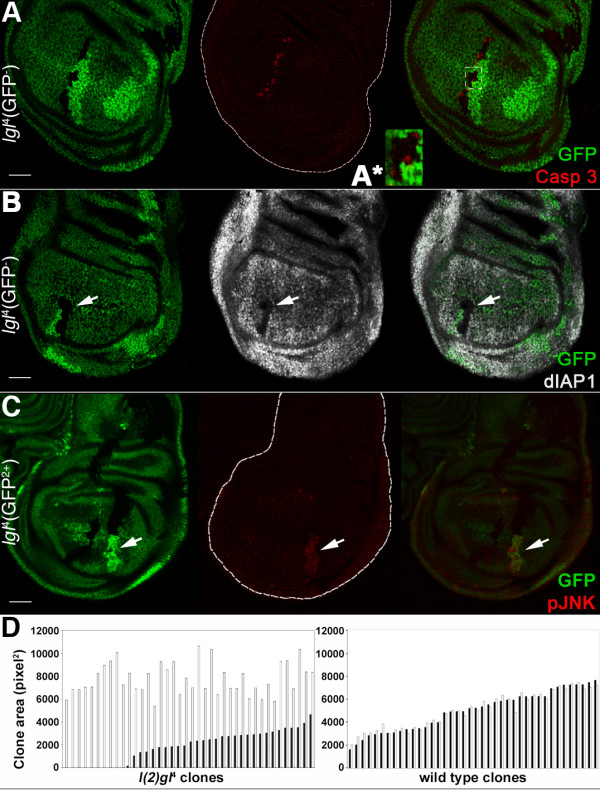
***lgl*^-/- ^clones induced in *lgl*^+/- ^imaginal wing discs die by apoptosis and the surrounding tissue grows at their expense**. **A, B**: *lgl*^-/- ^clones (GFP^-^) induced in a *w, hs-Flp*/+; *l(2)gl*^4^, FRT40A/*Ubi>GFPnls*, FRT40A background (GFP^+^). Wild-type twin clones are GFP^2+^. A: active-Caspase 3 staining; in A*, a magnification of the region outlined is shown. B: dIAP1 staining; its expression within the mutant clone (arrow) is visibly lower. **C**: *lgl*^-/- ^clones (GFP^2+^) induced in a *w, hs-Flp*/+; *l(2)gl*^4^, *Ubi>GFPnls*, FRT40A/FRT40A background (GFP^+^). Wild-type twin clones are GFP^-^. pJNK staining shows that the JNK pathway is activated inside the *lgl *mutant clone (arrow). Wing discs are outlined in A and C. Scale bars are 35 μm. Mutant clone genotypes are indicated. **D**: *lgl*^-/- ^(left panel) and wild-type (right panel) clone profile from a twin analysis of *l(2)gl*^4 ^or wild-type clones sampled in the wing pouch region induced in a *w, hs-Flp*/+; *l(2)gl*^4^, FRT40A or FRT40A/*Ubi>GFPnls*, FRT40A background. Black bars indicate *lgl*^4 ^(n = 40) and wild-type (n = 40) clones and white bars indicate the respective twins. For this experiment, freshly hatched larvae were collected in a one-hour time window and staged on cornmeal medium to 90 hours after hatching before collecting tissues.

In order to investigate the proliferation and polarity phenotypes of *lgl *mutant cells during development, we induced clones of two different *lgl *null mutations in *Drosophila *wild-type wing discs, larval proliferating epithelia where competitive mechanisms between genetically different cell populations have been extensively characterised [14,15]. As can be observed in Figure 1A and Figure S1A, B in Additional File 1, *lgl*^4 ^[21] or *lgl*^27S3 ^[22] mutant clones grew preferentially in the proximal-distal direction, in a pattern similar to that of wild-type clones [23]. At two days after induction *lgl*^-/- ^clones and their twin *lgl*^+/+ ^clones were similar in size (Figure S1A in Additional file 1), while at three days *lgl*^-/- ^clones were about one half the size of their twins (bright white, compare Figure S1B and Figure S1A in Additional file 1, n = 40 each) and many of them (37%, n = 287) disappeared by the end of larval development, leaving only the twin clone (Figure S1B in Additional file 1, arrow). However, some persisted to the end of development, resulting in scarring of the adult wing (Figure S1C in Additional file 1).

Staining for active-Caspase 3 revealed that *lgl*^-/- ^cells die by apoptosis (Figure 1A) and in basal sections many pycnotic nuclei were visible within *lgl*^-/- ^clones (Figure S1E in Additional file 1), confirming the presence of dying cells. Caspase activation occurred mostly in mutant cells at clonal boundaries, where mutant and normal tissues were in close contact (outlined, magnified in Figure 1A*). The anti-apoptotic protein *Drosophila *Inhibitor of Apoptosis 1 (dIAP1), ubiquitously expressed in imaginal tissues [24], was downregulated in *lgl*^-/- ^cells (Figure 1B, arrow), whereas the JNK pathway was activated (as detected by phospho-JNK staining) in the mutant clones (Figure 1C, arrow). Possible crosstalk among these pathways in inducing cell death in *lgl *mutant clones will be discussed in detail in Section 5.

**Figure 2:**
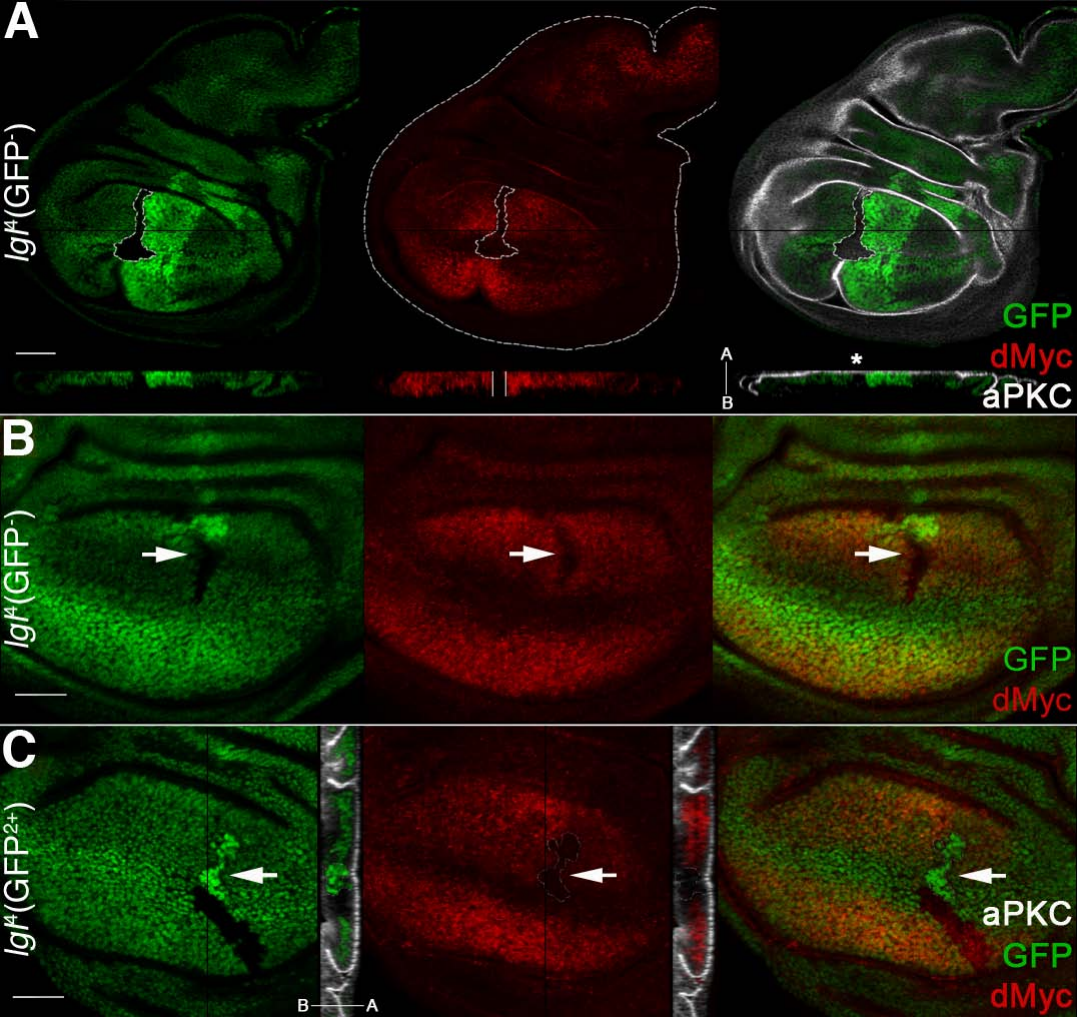
***lgl *mutant clones show low levels of dMyc oncoprotein with respect to the surrounding tissue**. **A, B**: *lgl*^-/- ^clones (GFP^-^) induced in a *w, hs-Flp*/+; *l(2)gl*^4^, FRT40A/*Ubi>GFPnls*, FRT40A background (GFP^+^). Wild-type twin clones are GFP^2+^. A: dMyc and aPKC staining; clone is outlined and the projection along the Z axis shows that dMyc expression within the mutant clone is low all along the disc thickness (enclosed between two white bars) and does not show strong defects in apical-basal cell polarity (asterisk). The apical-basal axis of the disc proper is also shown. Another *lgl*^-/- ^clone showing low dMyc levels (arrow) can be observed in B at higher magnification. **C**: To show mutant nuclei, *lgl*^-/- ^clones (GFP^2+^, arrow) were also induced in a *w, hs-Flp*/+; *l(2)gl*^4^, *Ubi>GFPnls*, FRT40A/FRT40A background (GFP^+^). Wild-type twin clones are GFP^-^. In the projection along the Z axis it can be seen that *lgl*^-/- ^cells are being basally extruded from the epithelium. Wing disc is outlined in A. Scale bars are 35 μm. Mutant clone genotypes are indicated.

*lgl *mutant clones showed slight defects in disc folding, as revealed by F-actin staining (Figure S1D in Additional file 1, arrow); however, mutant cells did not seem to be strongly compromised in apical-basal polarity (as detected by aPKC and Scribble staining), at least up to 72 hrs after clone induction (Figures 2C and S1F, G in Additional file 1), and no discontinuities in basement membrane were visible (not shown), in agreement with previous observations [22,25].

### *lgl *mutant cells express low levels of dMyc oncoprotein compared with the adjacent epithelium, and the latter grows at the expense of the *lgl*^-/- ^clones

To test whether competitive interactions were involved in the elimination of *lgl*^-/- ^cells from the normal tissue, we performed a double clonal assay in which *lgl*^-/- ^clones were induced in an *lgl*^+/- ^background in parallel to wild-type clones induced in a wild-type background (Figure 1D). As expected, while no differences were observed between wild-type control clones and their twins (right panel, black and white bars respectively, *P *= 0.86), *lgl *mutant clones were smaller than their wild-type twins (left panel, black and white bars respectively, *P *< 0.001), as well as smaller than wild-type clones induced in control discs (right panel, black bars, *P *< 0.001). The wild-type twins of *lgl*^-/- ^clones (left panel, white bars) were instead much larger than the wild-type twins from control discs (right panel, white bars, *P *< 0.001). Since no dominant effects on cellular growth rate have been reported for *lgl *null mutations, and developmental stages of *lgl*^+/- ^animals are of the same duration as those of *lgl*^+/+ ^individuals, even in clonal assays, this result suggests that some non-autonomous mechanisms are at work in *lgl*^-/- ^clone elimination and that mutant cells are being replaced by the normal surrounding tissue.

Since it is known that differences in dMyc abundance among adjacent cells can trigger competitive behaviour [14,15], we next analysed dMyc protein levels and found that dMyc was weakly expressed within *lgl *mutant clones with respect to the surrounding *lgl*^+/- ^tissue (Figure 2). We therefore speculated that cells bearing precancerous lesions that do not confer a survival or proliferative advantage relative to neighbouring cells, as with *lgl*^-/- ^cells that lack dMyc protein, can be eliminated by apoptosis. In the wing disc, dMyc protein accumulates mainly in the distal region, the wing pouch, and is expressed only weakly in the proximal regions, hinge and pleura (see Figure S2A in Additional File 1). In these proximal regions, no differences in dMyc levels were visible between *lgl*^-/- ^clones and the adjacent cells; such clones were larger than those in the wing pouch, although they never formed tumours (Figure S1G in Additional File 1. Proximal: 3,254 ± 1,129 pixels *vs *distal: 2,197 ± 756 pixels, n = 35 each; *P *< 0.001) and showed low levels of cell death (not shown). Thus, low dMyc levels in *lgl*^-/- ^cells seem to affect growth and viability of these cells mainly in the distal region (wing pouch), where the surrounding tissue expresses high levels of dMyc.

### dMyc overexpression within *lgl *mutant clones unleashes their neoplastic potential

**Figure 3:**
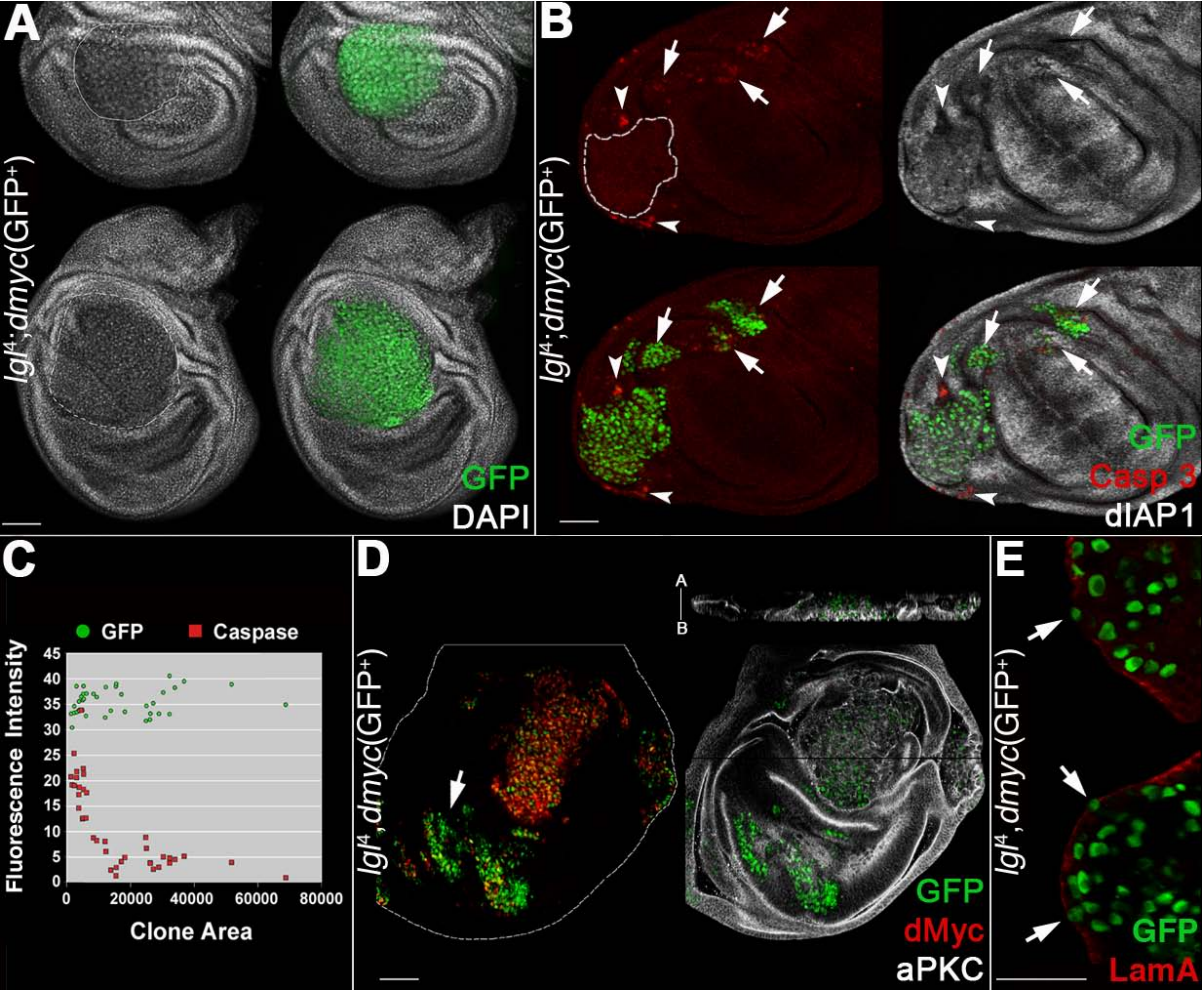
**dMyc expression in *lgl*^-/- ^clones induced in an *lgl*^+/- ^background promotes overgrowth and invasive behaviour**. **A-E**: *lgl*^-/-^; *UAS-dmyc *clones (GFP^+^) in a *yw*, *hs-Flp*, *tub>Gal4*, *UAS-GFP*/+; *l(2)gl*^4^, FRT40A/*tub>Gal80*, FRT40A; *UAS-dmyc*/+ background (GFP^-^). A: clone morphology. B: active-Caspase 3 and dIAP1 staining; the arrows indicate groups of mutant cells undergoing autonomous apoptosis and the arrowheads indicate *lgl*^+/- ^cells dying outside a mutant clone. C: distribution of the ratios 'clone area/active Caspase 3 fluorescence intensity' (red squares) and 'clone area/GFP fluorescence intensity' (green circles) as a control (n = 40); D: dMyc and aPKC staining; the projection along the Z axis shows the multilayered nature of the epithelium inside the mutant clone. The apical-basal axis of the disc proper is also indicated. The arrows indicate clones in the wing pouch that show low levels of dMyc protein. E: Laminin A staining; the arrows indicate regions of discontinuity. Wing disc is outlined in D. Scale bars are 35 μm. Mutant clone genotypes are indicated.

To investigate if the reduced level of dMyc protein in *lgl *mutant cells could be a cause of their elimination from the epithelium, we took advantage of the MARCM system [26] to express the *UAS-dmyc *construct [27] in *lgl*^-/- ^clones. As can be observed in Figure 3A, *lgl*^-/-^; *UAS-dmyc (dmyc*^over^) clones were round in shape and massively overgrown, with the largest clones located in the proximal regions of the disc. A 10-fold increase in clone area was observed with respect to *lgl*^-/- ^control clones induced through the same genetic system (average area of clones sampled in the hinge/pleura: 24,518 ± 4,237 pixels *vs *2,725 ± 1,146 pixels respectively, n = 68 each). A gain in cell size has been reported for *dmyc*^over ^cells [28], raising the question of whether this could contribute in a significant way to *lgl*^-/-^; *dmyc*^over ^clone area. Our analysis confirmed that cells in *lgl*^-/-^; *dmyc*^over ^clones were larger than in *lgl*^-/- ^control clones (83 ± 41 pixels vs 67 ± 32, n = 80 each), but this gain in cell size accounts only for a small fraction of clone expansion, indicating that the massive overgrowth of *lgl*^-/-^; *dmyc*^over ^clones is due mainly to a substantial increase in cell number.

dMyc ectopic expression is associated with an autonomous increase in apoptosis [29], so we expected to find high levels of cell death within *lgl*^-/-^; *dmyc*^over ^clones. However, while small clones showed many active-Caspase 3 positive cells (Figure 3B, arrows), the majority of the large clones showed little or no active-Caspase 3 staining (Figure 3B, clone outlined). We indeed found a significant negative correlation between clone size and active-Caspase 3 staining (Figure 3C, Spearman's rank correlation coefficient 0.716, *P *< 0.001). Furthermore, active-Caspase 3 staining was evident immediately outside the overgrown clones (Figure 3B, arrowheads), indicating that dMyc overexpression conferred *lgl*^-/- ^cells the ability to out-compete neighbouring cells. dIAP1 protein was expressed in the overgrowing mutant clones (Figure 3B), but was undetectable in the smallest clones, where the active-Caspase 3 signal is the strongest (Figure 3B, arrows).

*dmyc *overexpression in *lgl*^-/- ^cells therefore had a different outcome depending on the region of the disc where clones were located. In the proximal regions (hinge and pleura), where the endogenous dMyc levels are very low and competitive interactions between *lgl*^-/- ^and wild-type cells are relaxed, some *lgl*^-/-^; *dmyc*^over ^cells survived and formed clonal progeny that expressed dIAP1 and was therefore able to take advantage of dMyc's role in promoting biosynthesis and proliferation to out-compete surrounding cells (Figure 3B, arrowheads; see also the large clone in the hinge region in Figure 3D). *lgl*^-/-^; *dmyc*^over ^clones located in the distal region (pouch) instead grew poorly, did not express dIAP1 protein and underwent untimely cell death (Figure 3B, arrows); the explanation for this behaviour could be because endogenous dMyc is expressed at high levels here, which is likely to prevent these *lgl*^-/-^; *dmyc*^over ^clones from acquiring a competitive advantage. Other mechanisms could also be involved in generating such regional diversity in clonal growth, and further work is required to address this complex issue.

A staining for the apical marker aPKC indicated that *lgl*^-/-^; *dmyc*^over ^cells displayed impaired apical-basal polarity (Figure 3D, see in particular the Z projection), a typical hallmark of epithelial neoplasias [30]. Staining for Laminin A, a major component of the basement membrane in both *Drosophila *and mammalian epithelia, revealed signs of discontinuity across the mutant clones (Figure 3E, arrows), suggesting that *lgl*^-/-^; *dmyc*^over ^cells can acquire invasive properties, as has been demonstrated with other cooperative oncogenic models in *Drosophila *[25,31,32]. No pharate adults were recovered from larvae carrying *lgl*^-/-^; *dmyc*^over ^clones (not shown), indicating that the differentiation of adult structures was impaired.

As discussed above, *lgl*^-/-^; *dmyc*^over ^clones in the wing pouch behave differently from those in the wing hinge/pleura. In Figure 3D, the arrow indicates a clone in the wing pouch that does not exhibit tumourous properties. Notably, despite expression of *UAS-dmyc *transgene, clones do not show the high levels of dMyc protein observable in the clone located in the hinge. Since *dmyc *expression was induced using a heterologous promoter, the fact that dMyc protein is low in the *lgl *mutant clones in the wing pouch suggests that in this region an *lgl*-dependent regulation of *dmyc *at a post-transcriptional level is at work. Moreover, this result suggests that dMyc protein must be stably upregulated in *lgl *mutant clones in order to trigger overgrowth. This observation is similar to that of many human cancers, where Myc protein is highly and stably overexpressed [33]. Altogether, these data highlight a key role for *dmyc *in cooperating with the loss of a tumour suppressor to promote proliferation and to unleash the invasive potential of mutant cells.

### dp110 kinase expression fails to rescue the defective growth of *lgl *mutant cells

To understand whether dMyc could be substituted in rescuing the defective growth of *lgl*^-/- ^cells in an *lgl*^+/- ^background by other growth-promoting molecules, we expressed in *lgl*^-/- ^clones an activated form of the catalytic subunit of the Phosphoinositide-3 Kinase, *dp110*^CAAX ^[34], whose well-known role in cell growth and proliferation does not involve CC [14]. As can be observed in Figure S3B in Additional File 1, *dp110 *is not able to rescue the defective growth of *lgl*^-/- ^cells. Indeed, *UAS-dp110*^CAAX^; *lgl*^-/- ^clones are comparable in size to *lgl*^-/- ^clones induced through the same technique (average area 1,974 ± 753 pixels *vs *1,894 ± 916 pixels respectively, n = 40 each, *P *= 0.67); moreover, no significant impairments in cell polarity were observed (see Figure S3B in additional file 1, Z projection). As a control, a disc bearing *dp110*^CAAX ^clones is shown (Figure S3A in Additional file 1). Since it has been demonstrated that the ectopic expression of genes in the Insulin pathway, such as *dp110*, has no effect on ribosome biogenesis in *Drosophila *[35], it is plausible that the competitive properties of dMyc are due mainly to an increase in ribosome biogenesis [13,15]; its effect in increasing growth, proliferation and survival of *lgl *mutant cells could be due to the boosting of their biosynthetic rates.

### Inhibition of cell death is not sufficient for driving tumourous growth of *lgl *mutant clones in the wing pouch region

**Figure 4:**
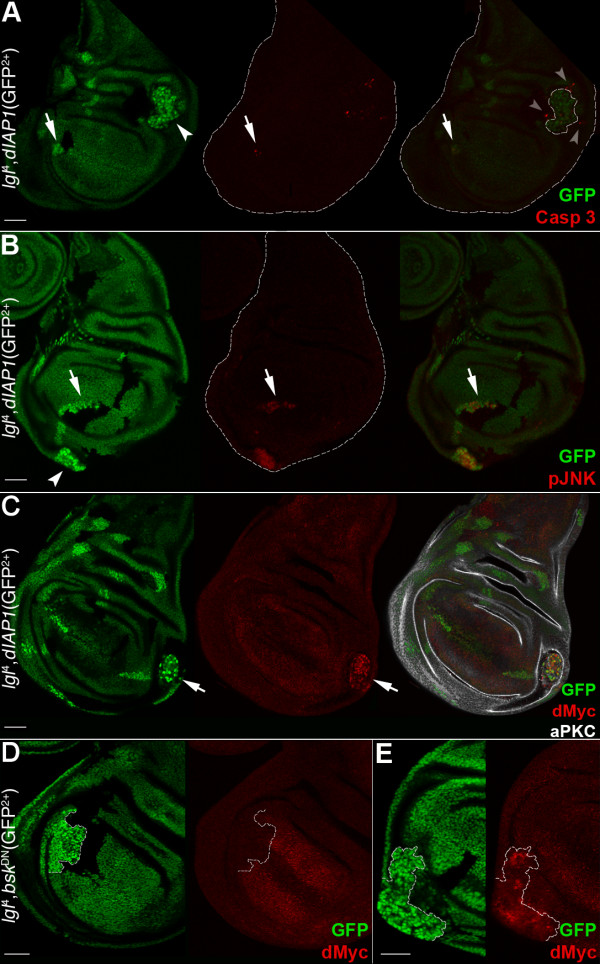
**Inhibition of cell death is not sufficient to unveil the malignant potential of the *lgl*^-/- ^clones in the wing pouch region**. **A-C: ***lgl*^-/-^; *UAS-dIAP *clones (GFP^2+^) induced in a *yw*, *hs-Flp*, *tub>Gal4*/+; *l(2)gl*^4^, *Ubi>GFPnls*, FRT40A/*tub>Gal80*, FRT40A; *UAS-dIAP*/+ background (GFP^+^). Wild-type twin clones are GFP^-^. Active-Caspase 3 (A) and pJNK (B) signals are both visible inside *lgl *mutant clones (arrows). Arrowheads indicate clones in the proximal regions and grey arrowheads point to active-Caspase 3 signals in *lgl*^+/- ^cells surrounding the mutant clone (outlined). In C, an *lgl*^-/- ^clone expressing high levels of dMyc protein is shown (arrow). **D, E**: *UAS-bsk*^DN^; *lgl*^-/- ^clones (GFP^2+^) induced in a *yw*, *hs-Flp*, *tub>Gal4/UAS-bsk*^DN^; *l(2)gl*^4^, *Ubi>GFPnls*, FRT40A/*tub>Gal80*, FRT40 background (GFP^+^). Wild-type twin clones are GFP^-^. dMyc protein is low in the mutant clone in the wing pouch (D) but is high in the clone in the pleura (E). Clone boundaries are indicated by the white dotted line. Wing discs are outlined in A and B. Scale bars are 35 μm. Mutant clone genotypes are indicated.

Since several molecules associated with cell death were found to be deregulated in *lgl *mutant clones generated in an *lgl*^+/- ^background (see Figure 1), we tried to rescue *lgl *mutant clones viability by co-expressing *dIAP1 *or a dominant negative form of the *Drosophila *JNK, *basket (bsk)*. As can be seen in Figure 4A, *lgl*^-/-^; *UAS-dIAP1 *clones in the wing pouch still showed active-Caspase 3 signals (arrow). A statistical analysis performed on clones found in the wing pouch indeed demonstrated that they are smaller than their wild-type twins (average area 7,721 ± 944 pixels *vs *11,583 ± 1,248 pixels respectively, n = 28; *P *< 0.001). Since the JNK pathway is activated in *lgl *mutant clones in the wing pouch (Figure 1C, arrow), it could significantly contribute to their death. It has however previously been reported that JNK activation also occurs in response to dIAP1 inactivation [36,37]; therefore if the reverse interaction occurred it may be expected that JNK signalling would be absent in *lgl*^-/-^; *UAS*-*dIAP1 *cells but, as shown in Figure 4B, there is strong upregulation of active JNK, as detected by pJNK staining (arrow), indicating that it contributes to the elimination of *lgl *mutant clones independently of dIAP1. Both pJNK and active-Caspase 3 were also visible in the mutant clones in the proximal regions of the wing disc (Figure 4A, B, hinge and pleura respectively, white arrowheads), but their phenotype was quite different from that of clones in the wing pouch; they were large, round-shaped and, as can be seen in Figure 4A, active-Caspase 3 signal was also present in several cells surrounding the mutant clone (grey arrowheads). A statistical analysis showed that such clones were larger than their wild-type twins (average area 15,243 ± 1,228 pixels *vs *9,855 ± 1,991 pixels respectively, n = 22; *P *< 0.01) These phenotypes were associated with high dMyc levels, as shown in Figure 4C (arrow); thus JNK signalling may here be subverted from a pro-apoptotic to a pro-growth function, as has been demonstrated to occur in genetic contexts in which alterations in the polarity genes *lgl*, *scrib *and *dlg *are accompanied by an ectopic expression of activated (oncogenic) Ras or Notch [32,38,39].

We then blocked the JNK pathway inside the *lgl*^-/- ^clones by using a *bsk*^DN ^transgene [39] and found that the active-Caspase 3 signal was no longer observable in *lgl*^-/- ^clones, regardless of the region in which they were located (not shown), indicating that JNK signalling-induced cell death is the main pathway by which *lgl *mutant cells die. As can be seen in Figure 4D, *UAS*-*bsk*^DN^; *lgl*^-/- ^clones in the wing pouch (outlined) expressed low levels of dMyc and did not form tumours; statistical analysis performed in this region showed that, despite the fact that *UAS*-*bsk*^DN^; *lgl*^-/- ^clones no longer died, their size was smaller than that of their wild-type twins (average area 12,697 ± 8,491 pixels *vs *21,957 ± 8,372 pixels respectively, n = 15, *P *< 0.001), demonstrating that even when cell death is blocked *UAS*-*bsk*^DN^; *lgl*^-/- ^cells have a proliferative disadvantage with respect to the wild-type tissue.

In contrast to the wing pouch, *UAS*-*bsk*^DN^; *lgl*^-/- ^clones in the proximal regions (hinge and pleura) showed high dMyc protein levels, lost polarity and overgrew (Figure 4E), indicating that the pro-growth role of the JNK pathway observed in polarity-compromised clones overexpressing activated Ras or Notch [32,38,39] appears not to be necessary in the wing disc for the tumourous growth induced by the cooperation between *lgl *mutation and dMyc oncoprotein. Altogether, these data show that the difference in clonal growth reported in section 2 for *lgl*^-/- ^cells in the wing pouch *versus *the hinge/pleura regions also occurs for *lgl*^-/- ^clones in which cell death has been inhibited and is also associated with the different levels of dMyc protein in the mutant *versus *the adjacent normal tissue.

### The Intrinsic Tumour Suppressor pathway does not appear to be involved in *lgl*^-/- ^cells elimination in the wing pouch region

**Figure 5:**
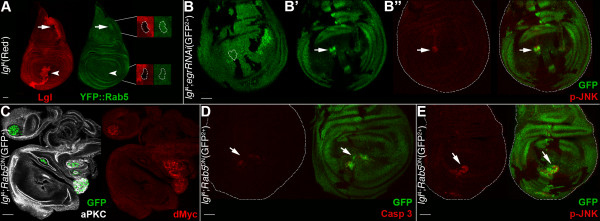
**Intrinsic Tumour Suppression is not responsible for *lgl*^-/- ^clonal death in the wing pouch region**. **A: **Lgl antibody staining (red) of *lgl*^-/- ^clones (black) induced in a *yw*, *hs-Flp*, *tub>Gal4/+*; *l(2)gl*^4^, FRT40A/*tub>Gal80*, FRT40; *tub>YFP::Rab5*/+background (red^+^). The wild-type twin clones are red^2+^. The arrow indicates an *lgl *mutant clone in the notum where the *YFP::Rab5 *signal is slightly increased and the arrowhead indicates an *lgl *mutant clone in the pouch where the *YFP::Rab5 *signal is comparable to that found in the surrounding *lgl*^+/- ^tissue. Magnifications of the mutant clones are shown on the right. **B-B": ***lgl*^-/-^;*UAS-egr*RNAi clones (GFP^2+^) induced in a *yw*, *hs-Flp*, *tub>Gal4*/+; *l(2)gl*^4^, *Ubi>GFPnls*, FRT40A/*tub>Gal80*, FRT40A; *UAS-egr*RNA *i*/+ background (GFP^+^). Wild-type twin clones are GFP^-^. B and B' show the apical and basal sections of the same disc; in the basal section a mutant clone contains cells that are basally excluded from the epithelium (arrow) and shows pJNK staining (B", arrow). The clone contour is outlined in B. **C:**, *lgl*^-/-^; *UAS-YFP::Rab5*^DN ^clones (GFP^+^) induced in a *yw*, *hs-Flp*, *tub>Gal4, UAS-GFP*/+; *l(2)gl*^4^, FRT40A/*tub>Gal80*, FRT40A; *UAS-YFP::Rab5*^DN^/+ background (GFP^-^), stained with aPKC and dMyc. Due to high staining intensity inside the mutant clones, dMyc signal has been kept very low to avoid overexposure, so its endogenous expression is barely visible. **D, E**: *lgl*^-/-^; *UAS-YFP::Rab5*^DN ^clones (GFP^2+^) induced in a *yw*, *hs-Flp*, *tub>Gal4*/+; *l(2)gl*^4^, *Ubi>GFPnls*, FRT40A/*tub>Gal80*, FRT40A; *UAS-YFP::Rab5*^DN^/+ background (GFP^+^). Wild-type twin clones are GFP^-^. D: active-Caspase 3; E: pJNK. Wing discs are outlined in B", D and E. Scale bars are 35 μm. Mutant clone genotypes are indicated.

A recent study demonstrated that clones of *scrib *and *dlg *tumour suppressor mutants generated in wild-type imaginal discs are eliminated by the Intrinsic Tumour Suppressor (ITS) pathway, involving JNK-dependent apoptosis induced by an endocytic accumulation of the TNF homologue, Eiger (Egr) [8]. Since *scrib *and *dlg *are well-known *lgl *partners in regulating apical-basal cell polarity and proliferation and show similar neoplastic phenotypes in a homotypic background [2], it could be expected that *lgl*^-/- ^clones could be eliminated by a similar mechanism. To investigate this, we first looked for alterations in endocytosis in the *lgl *mutant clones by using an early-endosome reporter, *Rab5*, since it was observed by Igaki et al. [8] that Rab5-positive endosomes accumulated in *scrib *mutant clones in the eye disc correlating with pJNK staining. However, in the wing pouch region we did not observe changes in Rab5 levels within *lgl*^-/- ^clones with respect to the neighbours (see Figure 5A, arrowhead, and respective magnification), whereas outside the wing pouch a moderate increase in Rab5 levels in *lgl*^-/- ^clones was observed (see Figure 5A, arrow, and respective magnification). Since Igaki et al. [8] demonstrated that ITS depends on an autocrine TNF signalling, we then silenced the TNF homologue, *egr*, in *lgl *mutant cells by expressing a *UAS*-*egr*RNAi construct (for validation see Figure S4A, B in Additional File 1) and scored for changes in clone morphology, but no alterations were observed relative to the *lgl*^-/- ^clonal phenotype (Figure 5B-B"). *lgl*^-/-^; *UAS-egr*RNAi clones in the wing pouch were comparable in size to *lgl*^-/- ^clones induced through the same system (average area pixels 2,954 ± 876 *vs *3,177 ± 1,028 pixels respectively, n = 19 each; *P *= 0,58). Similar effects were seen in *lgl *mutant clones in discs in which the *UAS-egr*RNAi construct was expressed under the control of the *hedgehog *promoter in the whole posterior compartment, thereby also removing Egr protein in the *lgl*^+/- ^background (not shown). In Figure 5B and 5B', the apical and basal sections of a wing pouch are shown in which an *lgl *mutant clone is being basally extruded (in Figure 5B the position of the mutant clone is outlined), which also showed increased pJNK staining (Figure 5B"). The fact that JNK signalling is increased here suggests that, in this case, JNK pathway activation is not triggered by *egr*-mediated ITS. We next expressed in *lgl *mutant clones a dominant negative form of *Rab5 *[40] to block endocytosis, since in the Igaki et al. study [8] it was shown that blocking endocytosis decreased JNK signalling and cell death of *scrib *mutant clones. Indeed, we found that some *lgl*^-/- ^mutant clones expressing *Rab5*^DN ^in the proximal regions overgrew (13 out of 27 scored, Figure 5C), while clones in the wing pouch never did. Again, *lgl*^-/-^; *UAS-Rab5*^DN ^clones (arrows) in the wing pouch were much smaller than the wild-type twins (see Figure S4C in Additional File 1) and expressed both active-Caspase 3 (Figure 5D, arrow) and pJNK (Figure 5E, arrow), distinct from what has been observed for *scrib *mutant clones in the eye disc, where *Rab5*^DN ^expression increased their growth [8]. Notably, all the overgrowing clones observed in the proximal regions of the wing disc were characterised by high dMyc protein levels (Figure 5C). Taken together, these data exclude *egr*-mediated ITS as the main mechanism responsible for *lgl *mutant clones elimination, at least in the wing pouch.

### *lgl*^-/- ^cells grown in a Minute background overexpress dMyc, display a competitive behaviour and form malignant tumours

**Figure 6:**
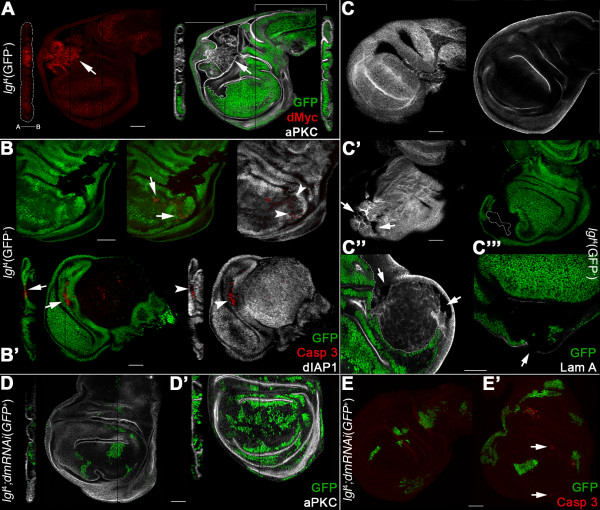
***lgl*^-/- ^malignant behaviour in a *Minute *background depends on dMyc protein levels**. **A-C"': ***lgl*^-/- ^clones (GFP^-^) in a *w, hs-Flp*/+;*l(2)gl*^4^, FRT40A/*M(2)24F, Ubi>GFPnls*, FRT40A background (GFP^+^). A: dMyc and aPKC staining. A strong dMyc accumulation is visible in the part of the mutant clone located in the hinge region (arrows). A Z projection of the mutant clone shows that loss of cell polarity accompanies the dramatic overgrowth; the apical-basal axis of the disc proper is shown. A Z projection of a region of the disc without mutant clones is shown on the right as a control. B: Activated Caspase 3 and dIAP1 staining. Arrows indicate *M/+, lgl*^+/- ^cells dying around the mutant clones; the Z projection confirms that dying cells are outside the *lgl*^-/- ^tissue. Arrowheads indicate that dying cells are deprived of dIAP1. C: Surface and cross sections of a wild-type disc stained with Laminin A. C'-C"': Laminin A staining of *lgl*^-/- ^clones in a *M/+, lgl*^+/- ^background; arrows indicate several points in which basement membrane integrity is lost. **D-E': ***lgl*^-/-^; *UAS-dm*RNAi clones (GFP^+^) in a *yw*, *hs-Flp*, *tub>Gal4*, *UAS-GFP*/+;*l(2)gl*^4^, FRT40A/*M(2)24F, tub>Gal80*, FRT40A; *UAS-dm*RNAi/+ background (GFP^-^). D, D': aPKC staining reveals that *lgl*^-/-^; *UAS-dm*RNAi mutant cells in a *M/+, lgl*^+/- ^background do not lose apical-basal polarity (compare with 6A). Heat shock pulses were 20 minutes (D) and 1 hour duration (D'). E, E': Caspase staining shows that apoptosis is either absent (E) or scattered throughout the disc (E', arrows). Scale bars are 35 μm. Mutant clone genotypes are indicated.

As we showed in section 5, *lgl*^-/- ^cells grew slower than wild-type cells in a clonal context, which is possibly the main reason why they are eliminated from the epithelium. To confirm the hypothesis for an active role of CC in restraining *lgl*^-/- ^clonal growth, we induced *lgl *mutant clones in the slow-dividing *Minute *background, to give them a proliferative advantage. As can be seen in Figure 6A-C"' and in the Additional File 2, *lgl *mutant clones in these larvae were able to overgrow (average area of clones sampled all across the wing disc: 12,974 ± 3,427 pixels *vs *2,648 ± 1,211 pixels of *lgl *mutant clones in a wild-type background, n = 40 each) and cells became rounded, indicating that apical-basal polarity was lost, as can be seen in the Z projection where aPKC apical determinant spreads cortically. *lgl*^-/- ^clones showed high levels of dMyc protein (Figure 6A, arrow), which accumulated mainly in those outside of the wing pouch (see also Figure S5 in Additional File 1). dMyc upregulation is attributable to *lgl *loss of function because wild-type cells did not show any changes in dMyc levels when growing in a *M/*+ background (Figure S6 in Additional file 1; in particular, no dMyc accumulation was visible in a large clone in the hinge region, S6A, arrow). *lgl*^-/- ^clones in the wing pouch did not overgrow, showed a level of dMyc similar to that found in the *M/*+, *lgl*^+/- ^adjacent cells (Figure S5A in Additional File 1, clone outlined) and did not undergo apoptosis (not shown). In that region, dMyc endogenous expression is high (see Figure S5A in Additional File 1), so it is plausible that its upregulation by the loss of function of *lgl *was not sufficient to trigger the cell growth advantage necessary for driving clonal expansion at the expense of the adjacent tissue.

In contrast, in regions where dMyc abundance is very low, such as the hinge and the pleura, dMyc upregulation by *lgl *mutation might be sufficient for clone overgrowth. In large *lgl*^-/- ^clones in the hinge or pleura, dIAP1 was also expressed (Figure 6B') and active-Caspase 3 signal was evident in few cells in the mutant clones, but marked mainly groups of surrounding cells (Figure 6B, B', arrows), which were deficient in dIAP1 (Figure 6B, B', arrowheads). Moreover, cells lost polarity (Figure S5 in Additional file 1) and the basement membrane showed signs of discontinuity (arrows in Figure 6C'-C"', compared with the wild-type disc shown in C), indicating invasive behaviour. No pharate adults were recovered from mosaic larvae (not shown). Altogether, these data show that *lgl*^-/- ^cells grown in a *M/*+, *lgl*^+/- ^background can acquire a competitive advantage, which correlates with high dMyc expression and polarity loss. The upregulation of dMyc protein inside *lgl*^-/- ^clones induced in the *M/*+ background may be a consequence of an increase in *dmyc *transcript levels, since an upregulation of *dmyc *transcription, assayed by a *dm>LacZ *construct [41] (*dm *stands for *diminutive*, the gene encoding dMyc protein) was observed (Figure S7B in Additional File 1). In contrast, there was no correlation between *dmyc *transcription and protein abundance in *lgl*^-/- ^clones grown in the *lgl*^+/- ^background; the reduced levels of dMyc protein observed (Figure 2) did not result from a decrease in *dmyc *transcription (Figure S7A in Additional File 1), therefore post-transcriptional mechanisms must be at work in this case.

To determine the functional importance of dMyc upregulation in *lgl*^-/- ^clones induced in the *M/*+, *lgl*^+/- ^background, we silenced *dmyc *inside *lgl*^-/- ^clones using a *UAS-dm*RNAi construct (for validation, see Figure S2C in Additional File 1). Knockdown of *dmyc *expression in *lgl*^-/- ^clones prevented the cell polarity defects observed with *lgl*^-/- ^clones alone (Figure 6D, compared with Figure 6A-C, Z projections), even when the *lgl *mutant tissue occupied a larger proportion of the disc (Figure 6D'); no degradation of basement membrane occurred (not shown) and clones did not form tumourous masses, regardless of the region in which they were located. Adults were indeed recovered at the expected frequency (not shown). Concerning the cell death pattern in these clones, it ranged from a complete absence of active-Caspase 3 signal in the majority of discs analysed (Figure 6E) to scattered signals, either in *lgl*^-/- ^cells, surrounding cells, or in regions distant from mutant clones (Figure 6E', arrows). These results indicate that *lgl*^-/-^; *UAS*-*dm*RNAi and *M/*+, *lgl*^+/- ^cells coexist in the same tissue without undertaking competitive interactions, possibly because both populations show a similar impairment in cell proliferation. Similar results were obtained by Wu and Johnston upon the induction of *dm *loss-of-function clones in a *M/*+ background [42]. *M/*+ tissue could also intrinsically possess a low level of dMyc protein, but we were not able to detect differences in dMyc protein levels between wild-type and *M/*+ clones throughout the wing disc (see Figure S6 in Additional File 1). Thus, the silencing of *dmyc *in *lgl*^-/- ^clones and the reduced ribosomal pool in the *M/*+, *lgl*^+/- ^background may make their levels of biosynthesis comparable. The ability of *lgl *mutant patches to grow despite dMyc deprivation might be due to the upregulation of other growth-promoting factors in the *M/*+, *lgl*^+/- ^context, which are however *per se *unable to provide *lgl*^-/- ^cells with tumourigenic features.

### The oncogenic cooperation between *lgl *mutation and dMyc protein is not tissue-specific

**Figure 7:**
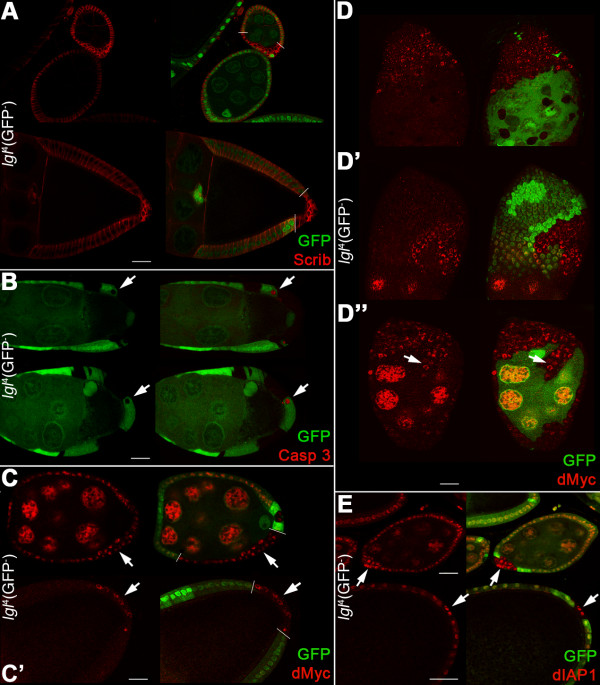
***lgl*^-/- ^clonal overgrowth is sustained by dMyc expression also in the follicular epithelium**. **A-E: **Twin-clone analysis of *lgl*^-/- ^clones (GFP^-^) induced in a *w, hs-Flp*/+; *l(2)gl*^4^, FRT40A/*Ubi>GFPnls*, FRT40A background (GFP^+^). Wild-type twin clones are GFP^2+^. A: *lgl*^-/- ^clones (bordered by white bars) stained for Scrib are shown in early and late egg chambers. Scrib distribution spreads from lateral to cortical as mutant cells become round. B: active-Caspase 3 staining of Stage 8 to 9 egg chambers; arrows indicate wild-type cells undergoing apoptosis. C: dMyc staining of early (C) and late (C') egg chambers. Bars mark clone boundaries and arrows indicate dMyc expression inside the mutant clones. D-D": dMyc staining in older *lgl*^-/- ^clones; in D" the arrow indicates mutant cells migrating into the nurse cell territory. E: dIAP1 staining of early and late egg chambers. The arrows indicate high dIAP1 expression in the *lgl*^-/- ^clones. Mutant clone genotypes are indicated.

With the aim of assessing whether the oncogenic cooperation between *lgl *mutation and dMyc protein could be conserved in other *Drosophila *tissues, we investigated *lgl*^-/- ^clonal behaviour in the ovarian follicular epithelium, a monolayered adult tissue of somatic origin that surrounds the germ line of the egg chamber. Interestingly, the phenotype of *lgl*^-/- ^clones in the follicular epithelium appeared to be rather different from that observed in the wing imaginal epithelia, but similar to that previously reported in the whole mutant animal; in females bearing a homozygous temperature-sensitive *lgl *mutation, egg chambers invariantly show hyperproliferation of follicular cells that display loss of apical-basal polarity and migrate between the nurse cells [43]. We found that *lgl *mutant clones overproliferated and formed multilayers near the chamber poles (Figure 7A), consistent with previous studies [2,44]. Active-Caspase 3 staining revealed that apoptosis was occasionally observable in egg chambers from Stage 8 onward in *lgl*^+/- ^cells at the clone border (Figure 7B). This cell death pattern is reminiscent of a mechanism of dMyc-induced CC. CC, however, has never been described in the follicular epithelium, so we induced *dmyc*^over ^Flp-out clones [45] in adult females and observed the cell death pattern in their egg chambers. As can be seen in Figure S8 in Additional File 1, the wild-type tissue underwent massive cell death, particularly when it was completely surrounded by *dmyc*^over ^cells (arrows), suggesting that *dmyc *is able to induce apoptosis in the surrounding cells also in this tissue. Indeed, *lgl*^-/- ^clones expressed dMyc protein in early egg chambers (Figure 7C) as well as at later stages (Figure 7C'), where endogenous protein is normally absent (see Figure S2B in Additional file 1). *dmyc *was also transcriptionally activated, as can be seen from *dm>LacZ *expression in Figure S7C in Additional file 1. Thus, *lgl*^-/- ^clonal behaviour in the follicular epithelium positively correlates with *dmyc *expression. dIAP1 levels in *lgl*^-/- ^follicular clones were similar to those seen in the *lgl*^+/- ^adjacent tissue (see Figure 7E), and no autonomous apoptosis was visible in *lgl*^-/- ^follicular cells (not shown). Further, we allowed clones to grow for four additional days in order to obtain stronger phenotypes. As can be seen in Figure 7D-D", dMyc protein was detectable in all *lgl*^-/- ^cells; clonal phenotype varied from larger areas of multilayered tissue (Figure 7D), which sometimes extended away from the poles (Figure 7D'), to clones that invaded into the nurse cells territory (Figure 7D", arrow). To determine the contribution of *dmyc *to the *lgl *mutant phenotype in this tissue, we lowered dMyc levels inside *lgl*^-/- ^cells using the *UAS*-*dm*RNAi construct. We observed a pronounced decrease both in clone size and number (visible clone area: 44% decrease in 60 clones at chamber poles analysed for each genotype; clone number: 74% decrease on a total of 20 pairs of ovaries for each genotype) and no multilayered tissue was seen, demonstrating that also in this epithelium dMyc protein is required for *lgl *mutant clones to grow as malignant tumours.

## Conclusions

Deciphering the molecular crosstalk occurring between genetically different cell populations growing within a tissue is relevant to both developmental and cancer biology. In *Drosophila*, *lgl *mutant larvae show neoplastic overgrowth of the epithelial tissues, whereas *lgl*^- ^cells growing in mosaic larvae are usually eliminated by cell death; thus this opposite phenotype results from mechanisms triggered by the confrontation of *lgl*^- ^and *lgl*^+ ^cells. In the literature, two main processes have been described to mediate the elimination of unfit/polarity-deficient cells from *Drosophila *imaginal epithelia: *Cell Competition *(CC) and *Intrinsic Tumour Suppression *(ITS) [9]. Both mechanisms work by the induction of cell death in the mutant population; in addition, normal tissue grows at the expense of the unfit cells in CC [15], while this has not been shown for ITS [8]. There is suggestive evidence in the literature regarding the relevance of CC to cancer biology [12,13,46], however a clear connection between CC and tumour development is yet to be made.

dMyc oncoprotein has been shown to play a leading role in CC, in the sense that cells bearing low levels of this protein die by apoptosis if growing in a high dMyc-expressing context [14,15]. In ITS, cell death is instead triggered in the presence of cells with impaired polarity in an otherwise normal epithelium [8]. Here we found that JNK-mediated apoptosis is the main process through which *lgl *mutant clones are eliminated from imaginal wing epithelia (Figures 1C and 4D). Clonal phenotype is however different in the distal (wing pouch) and proximal (hinge/pleura) regions of the wing disc: *lgl *mutant clones located in the wing pouch showed massive cell death and were rapidly extruded from the epithelium (Figures 1 and S1E-F in Additional file 1), while clones located in the hinge/pleura grew to a larger extent, although they never formed tumours (Figure S1G in Additional file 1). This regional difference in clonal behaviour could depend on dMyc protein pattern in the wing disc; the protein is highly expressed in the wing pouch and very low in the hinge/pleura (Figure S2A in Additional file 1). We indeed found that *lgl*^- ^clones in the wing pouch expressed very low levels of dMyc protein relative to the background (Figure 2); such clones died and were replaced by the surrounding wild-type tissue (Figure 1D). Moreover, *lgl*^- ^clonal behaviour in the slow-dividing *Minute *background (Figure 6) or in the follicular epithelium (Figure 7) switched from *out-competed *to *overgrown and invasive*, with the tumourous mutant clone invariantly showing high dMyc levels relative to the neighbouring tissue. Lowering dMyc abundance inside those clones prevented the tumourous phenotype (Figure 6D and data not shown), whereas dMyc overexpression in *lgl*^- ^mutant clones in a wild-type background was able to confer increased proliferation, survival and invasive properties (Figure 3), clearly demonstrating that dMyc plays a key role in *lgl*^- ^cells behaviour. In all the cases analysed, clonal overgrowth of *lgl*^- ^*dmyc*^over ^tissue was observed in the proximal regions of the wing disc, where endogenous dMyc is poorly expressed, underlining the importance of a favourable dMyc gradient between the mutant clone and the surrounding tissue in triggering *lgl*-induced malignancy. We also showed that ITS is not responsible for *lgl *mutant cells elimination in the wing pouch, in contrast to what was observed for the other two neoplastic tumour suppressor mutants *scrib *and *dlg *[8]; *lgl*^- ^clones located in the distal regions still died even upon *egr *silencing or endocytosis inhibition (Figure 5B-D). In the pouch, where dMyc protein is highly expressed, *lgl*^- ^cells instead underwent apoptosis due to low levels of dMyc protein relative to their neighbours, since when dMyc levels were restored in the mutant clones, as happens in a slow-growing *Minute *background, *lgl *mutant cells no longer died. This is first evidence that dMyc-induced CC protects tissues from tumourous growth. On the other hand, blockade of endocytosis in *lgl *mutant tissue located outside of the wing pouch resulted in clonal overgrowth, indicating that ITS is likely to be involved in the elimination of *lgl *mutant cells in this region. Also in this case, dMyc upregulation was observed in the overgrown mutant tissue (Figure 5C).

The main conclusion of our work is that dMyc protein levels dictate the behaviour of *lgl*^- ^tissue: when dMyc is highly expressed it cooperates with *lgl *mutations in a cell-autonomous manner, driving malignant growth in different contexts and organs, whereas when dMyc is downregulated in *lgl*^- ^tissue relative to the wild-type surrounding cells, the *lgl *mutant cells are eliminated by CC. Moreover, it appears that CC and ITS play an important role in epithelial integrity, eliminating potentially harmful cells in complementary regions of the wing disc.

How does Lgl depletion lead to dMyc upregulation in particular tissues? Recent analysis [47] has revealed that depletion of Lgl in the eye disc leads to deregulation of the Salvador-Warts-Hippo (SWH) tumour suppressor pathway, an evolutionarily conserved signalling pathway that controls organ size and prevents hyperplastic diseases from flies to humans by restricting the activity of the transcriptional coactivator Yorkie [48]. Further to this, we found that *dmyc *is a transcriptional target of the SWH pathway, and dMyc protein is strongly upregulated in mutant clones of many members of the pathway (unpublished data). Consistent with the SWH pathway being deregulated, the *bona fide *Yorkie target, dIAP1 [49,50] was upregulated in larger *lgl*^- ^clones that overgrew and expressed dMyc (Figure 6B' and data not shown), although further analysis of this in wing disc clones using other SWH targets is required to confirm this. We also note that the upregulation of dMyc in *lgl*^- ^tissue correlates with a loss in apico-basal polarity (Figures 6A and S5B in Additional file 1, arrow). Although in the eye disc *lgl*^- ^clones deregulate the SWH pathway without polarity loss [47], this does not seem to be the case in the wing, since the SWH target dIAP1 was only upregulated in *lgl*^- ^tissue where polarity is lost. The results of our study suggest that upon Lgl depletion, to the extent that apical-basal cell polarity is compromised, that is, in a *Minute *context in the wing disc or in the ovarian follicular cells, deregulation of the SWH pathway occurs and, as a consequence, upregulates *dIAP1 *and *dmyc*. The activity of the *dmyc *promoter is indeed increased in *lgl*^- ^clones induced in a *Minute *background and in follicular cells (Figure S7B, C in Additional file 1). In *lgl*^- ^clones induced in a wild-type context, where *lgl *mutant cells do not lose polarity (Figure S1F, G in Additional file 1), *dmyc *transcription is not altered (Figure S7A In Additional file 1). In these clones, dMyc protein is downregulated, which could occur by post-transcriptional mechanisms affecting protein stability [51].

Examples of these complex behaviours are also present in the literature describing mammalian carcinogenesis, in which the role of polarity proteins is still not well understood. It has been recently shown that *c-myc *cooperates with *scribble *tumour suppressor to induce the neoplastic progression observed during mammary tumourigenesis [52]. On the other hand, in a murine model of FAP, *APC*^- ^cells deprived of *c-myc *were out-competed by surrounding, *c-myc-*expressing wild-type cells, thereby reverting the malignant phenotype, suggesting that CC could play a role in mammalian carcinogenesis [53].

Complex molecular interactions among genetically different cells growing in an organ emerge from our study, which in turn activate several safeguard mechanisms to restrict overgrowth and preserve epithelial integrity. Due to the conservation of signalling pathways, cell proliferation, and cell death pathway genes between fly and humans, *Drosophila *represents an ideal system for analysing the early events occurring in tumourigenesis upon the confrontation of different cell populations, and further analysis will help dissect these mechanisms.

## Methods

### Mosaic analyses

For the list of mutant and transgenic flies utilised, please see Additional File 1.

*lgl*^27S3 ^and *lgl*^4 ^mutant phenotypes are fully rescued by expression of *UAS-lgl *transgenes, resulting in normal larval and adult structures [22,5].

For mosaic analyses, either the Flp-FRT [54] or MARCM [26] systems were used. For larval staging, freshly hatched larvae were collected in a one-to-four hour time window. For the twin analysis, larvae were heat shocked 48 hours After Egg Laying (AEL) for one hour at 37°C and adult females were heat shocked one to two days after eclosion for one hour at 37°C. For the MARCM system, larvae were heat shocked 48 hours After Egg Laying (AEL) for 20 minutes at 37°C. *Minute *individuals were always heat shocked at 72 hours AEL to compensate for developmental delay. For the Flp-out system [45], adult females were heat shocked one to two days after eclosion for eight minutes at 37°C. For all the systems used, tissues were collected 72 hours after the heat shock both for larvae and adults, unless otherwise specified. All crosses were kept at 25°C.

### Immunofluorescence

Imaginal discs and ovaries were fixed and stained according to standard protocols. The following antibodies and dilutions were used: rabbit anti-active-Caspase 3 (1:200, (9664S) Cell Signaling Technology, Inc., Danvers, MA, USA); mouse anti-dIAP1 (1:100, BA Hay); rabbit anti-Laminin A (1:200, Y Kitagawa); rabbit anti-Scribble (1:100, CQ Doe); mouse anti-dMyc (1:5) [55]; mouse anti-βGal (1:25, [40-1a] DSHB, University of Iowa, Iowa City, IA, USA); rabbit anti-βGal (1:50, F Graziani); rabbit anti-aPKCζ (1:200, [sc-216] Santa Cruz Biotechnology, Inc., Santa Cruz, CA, USA); mouse anti-phospho-JNK (1:100, [G9 clone] Cell Signaling Technology Inc., Danvers, MA, USA); rabbit a-Egr (1:500, M Miura). Alexa Fluor 555 or 568 goat anti-mouse and anti-rabbit (1:200, Invitrogen Corporation, Carlsbad, CA, USA) and Cy5-conjugated goat anti-mouse and anti-rabbit (1:100, Jackson ImmunoResearch Laboratories, Inc., West Grove, PA, USA) were used as secondary antibodies. DAPI and TOPRO staining were used to detect DNA and phalloidin staining to detect F-actin. Samples were analysed by laser confocal microscopy (Leica TSC SP2, Leica Microsystems SpA, Milan, Italy) and entire images were processed with Adobe Photoshop software. ImageJ free software from NIH, Bethesda, MD, USA was used for rebuilding the projections along the Z axis starting from 25-45 Z stacks.

### Measurements and statistical analysis

To measure clone area (in pixel^2^) and fluorescence intensity (in calibrated units), ImageJ free software from NIH, Bethesda, MD, USA, was used. Fluorescence intensity (signal intensity) was calculated as the average gray value within the selection. The average and the standard deviation alone or the t-Student test *P *value are given in the text. For the correlation between clone size and active-Caspase 3 staining in Figure 3, the Spearman's rank correlation coefficients were calculated using the software XLSTAT (available at http://www.xlstat.com). To measure average cell area in wing disc clones, TOPRO stained nuclei and clone area were scored in confocal series and the relative cell area (in pixels^2^) was calculated.

## Abbreviations

AEL: After Egg Laying; APC: Adenomatous Polyposis Coli; aPKC: atypical Protein Kinase C; bGal: beta Galactosidase; Bsk: Basket; Baz: Bazooka; CC: Cell Competition; Crb: Crumbs; DAPI: 4',6-Diamidino-2-Phenylindole; Dlg: Discs large; dIAP1: drosophila Inhibitor of Apoptosis 1; dm: diminutive; DN: Dominant Negative; Egr: Eiger; FAP: Familial Adenomatous Polyposis; Flp: Flippase; FRT: Flippase Recognition Targets; GFP: Green Fluorescent Protein; Hid: Head Involution Defective; hrs: hours; hs: heat shock; Hugl-1: Human giant larvae 1; ITS: Intrinsic Tumour Suppression; JNK: Jun N-terminal Kinase; Lgl: Lethal giant larvae; M: Minute; MARCM: Mosaic Analysis with a Repressible Cell Marker; nls: nuclear localisation signal; Over: Overexpressed; PATJ: Pals 1-Associated Tight Junction protein; PAR3: Partitioning defective 3; PAR6: Partitioning defective 6; Scrib: Scribble; Sdt: Stardust; UAS: Upstream Activating Sequences; vs: versus; pJNK: phosphoJNK; TNF: Tumour Necrosis Factor; RNAi: RNA interference; SWH: Salvador Warts Hippo; tub: tubulin; Ubi: Ubiquitin; YFP: Yellow Fluorescent Protein.

## Authors' contributions

FF, MZ and NAG carried out the experiments; FG participated in conceiving the project. PB, DS, HER, AP and AP* participated in designing the experimental layout and DG conceived the study and wrote the paper.

## Supplementary Material

Additional file 1 **Fly stocks and additional figures and legends in Portable Document Format**. Stock list and Eight additional figures with respective legends. Click here for file

Additional file 2 **Movie**. Movie of a *M/+ *imaginal wing disc in which a large *lgl *mutant clone (GFP-) is visible. The whole thickness of the disc is shown in which the overgrowth of the mutant clone is clearly observable. Click here for file
