# Supplementary material for: The lethal giant larvae tumour suppressor mutation requires dMyc oncoprotein to promote clonal malignancy
Source: BMC Biol. 2010 Apr 7;8:33. doi: 10.1186/1741-7007-8-33 (PMC2877678; doi:10.1186/1741-7007-8-33)
Supplement: Additional file 1 — Fly stocks and additional figures and legends in Portable Document Format. Stock list and Eight additional figures with respective legends. [file 1741-7007-8-33-S1.PDF]

The *lethal giant larvae* tumour suppressor mutation requires dMyc oncoprotein to promote clonal malignancy

Francesca Froldi[1,2], Marcello Ziosi[1,2], Flavio Garoia[3], Andrea Pession[4], Nicola A. Grzeschik[5], Paola Bellosta[6], Dennis Strand[7], Helena E. Richardson[5,8], Annalisa Pession[1]* and Daniela Grifoni[1,2]*§

1 Alma Mater Studiorum, Dipartimento di Patologia Sperimentale, Via S. Giacomo 14, 40126 Bologna, Italy.

2 Alma Mater Studiorum, Dipartimento di Biologia Evoluzionistica Sperimentale, Via Selmi 3, 40126 Bologna, Italy.

3 NGB Genetics s.r.l, University of Ferrara, Via Borsari 46, 44100 Ferrara, Italy.

4 Alma Mater Studiorum, Dipartimento di Ginecologia, Ostetricia e Pediatria, Via Massarenti 9, 40138 Bologna, Italy.

5 Peter MacCallum Cancer Centre, Research Division, 7 St. Andrew's Place, East Melbourne, Victoria, 3002, Australia.

6 City College of the City University of NY, Department of Biology, Convent Ave at 138th New York, NY 10031, USA.

7 Johannes Gutenberg University, First Department of Internal Medicine, 63 Obere Zahlbacherstr., 55131 Mainz, Germany.

8 Department of Anatomy and Cell Biology and Department of Biochemistry and Molecular Biology, University of Melbourne, Parkville, Victoria, 3052, Australia.

* These authors share senior authorship

§ Corresponding author

Fly stocks

The following mutant and transgenic fly strains were used:

w; *FRT40A*

w; His2A>GFP

w; UAS-dmyc [27]

w, hs-Flp; *FRT40A*

w; l(2)gl^{27S3} [22], *FRT40A / In(2LR)*

w, hs-Flp; arm>LacZ, FRT40A

w, hs-Flp; Ubi>GFPnls, FRT40A

w; l(2)gl^{4} [21], *FRT40A / In(2LR)*

w; l(2)gl^{4}, *Ubi>GFPnls, FRT40A / In(2LR)*

w; l(2)gl^{4}*, FRT40A / In(2LR); UAS-dmyc*

w; l(2)gl^{4}*, FRT40A / In(2LR); UAS-dm*RNAi (VDRC line 2947)

yw, hs-Flp, tub>GAL4; tub>GAL80, FRT40A

yw, UAS-dp110^{CAAX} [34]*; FRT40A / In(2LR)*

yw, UAS-dp110^{CAAX}*; l(2)gl*^{4}, *FRT40A / In(2LR)*

w; l(2)gl^{4}*, FRT40A / In(2LR); tub>YFP::Rab5* (LA Baena-López)

yw, hs-Flp; act5c>CD2>GAL4, UAS-GFP / TM6b

yw, hs-Flp; M(2)24F, Ubi>GFPnls, FRT40A / SM5

yw, hs-Flp, tub>GAL4, UAS-GFP; tub>GAL80, FRT40A

yw, UAS-bsk^{DN} [39]*; l(2)gl*^{4}, *Ubi>GFPnls, FRT40A / In(2LR)*

w; l(2)gl^{4}, *Ubi>GFPnls, FRT40A / In(2LR); UAS-dIAP1* (Bloomington 6657, Hay's lab)

*w; Ubi>GFPnls, FRT40A / In(2LR); UAS-egr*RNAi (VDRC line 45252)

w; l(2)gl^{4}, *Ubi>GFPnls, FRT40A / In(2LR); UAS-egr*RNAi

w; l(2)gl^{4}, *Ubi>GFPnls, FRT40A / In(2LR); UAS-YFP::Rab5*^{DN} [40]

yw, hs-Flp, tub>GAL4, UAS-GFP; M(2)24F, tub>GAL80, FRT40A / SM5

w, P[LacW]l(1)G0354 (*dm>LacZ*^{G0354}) [41]/ *yw, FM7a; l(2)gl*^{4}*, FRT40A / In(2LR)*

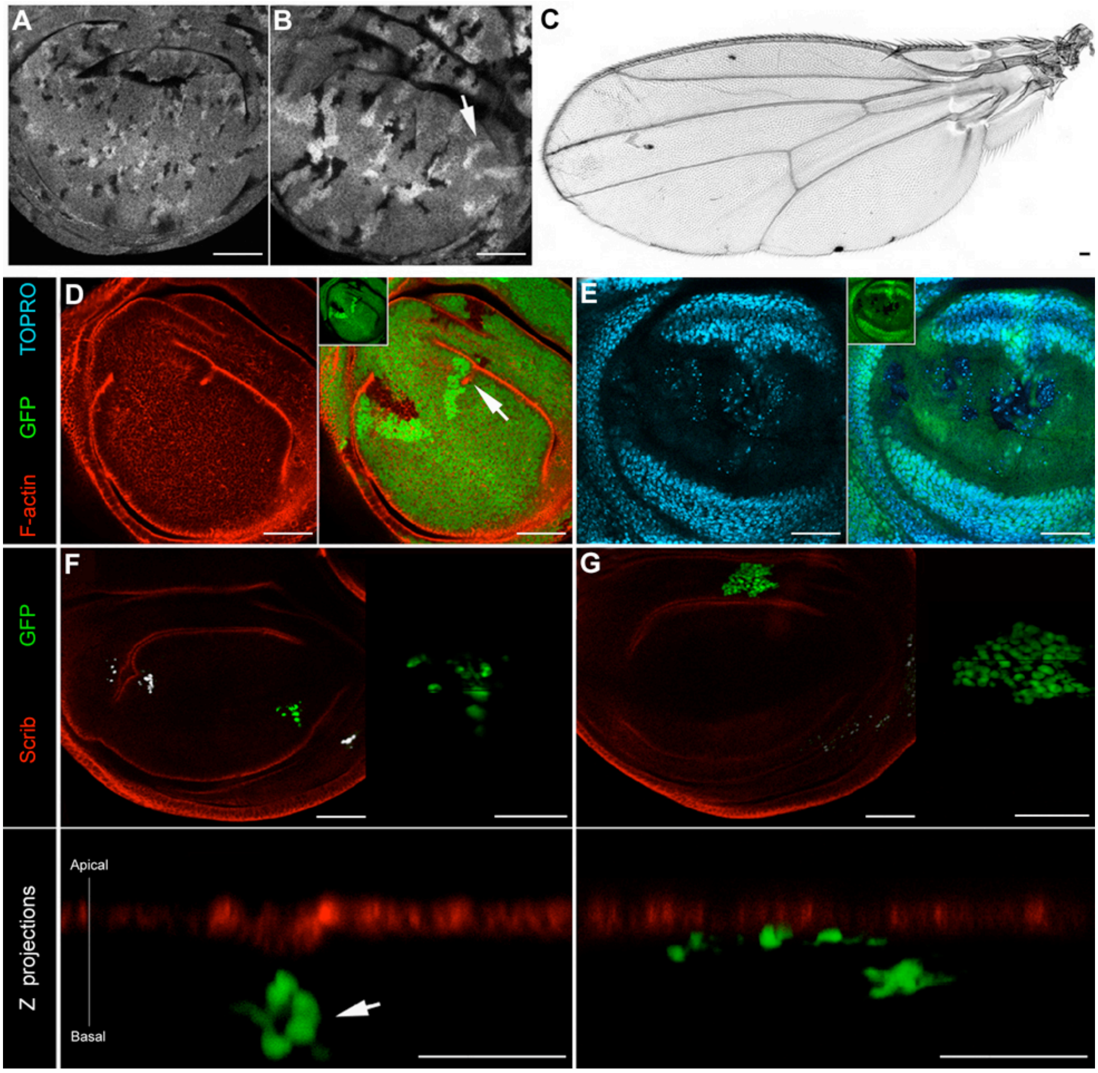

Figure S1. *lgl*$^{-/-}$ cells growing in a *lgl*$^{+/-}$ background do not show impairments in cell polarity during development

A,B: 2 (A) and 3 days-old (B) *lgl*^{27S3} clones (black) in a *w, hs-Flp/+; lgl*^{27S3}, FRT40A/*arm-LacZ*, FRT40A background (white^{+}). In B, the arrow indicates a twin clone (white^{2+}) with no *lgl*$^{-/-}$ counterpart. C: adult wing from a female of the same genotype showing apoptotic scars. D,E: apical (D) and basal (E) sections of *w, hs-Flp/+; lgl*^{27S3}, FRT40A/*Ubi>GFP*, FRT40A wing discs; in D, F-actin staining shows there is folding at the clonal boundaries (arrow); in E, pycnotic nuclei inside *lgl*^{27S3} clones (GFP^{-}). F,G: Scrib staining of wing discs bearing *lgl*^{4} clones in a *w, hs-Flp/+, tub>Gal4, UAS-GFP; l(2)gl*^{4}*,* FRT40A/*tub>Gal80,* FRT40A background. In the lower panel, the respective projections along the Z axis are shown; no changes in localisation of the subapical marker Scrib are appreciable in the *lgl*^{4} cells (GFP^{+}) with respect to the surrounding *lgl*$^{+/-}$ tissue (GFP^{-}), even when mutant cell nuclei are being basally extruded (arrow). Scale bars are 35 μm.

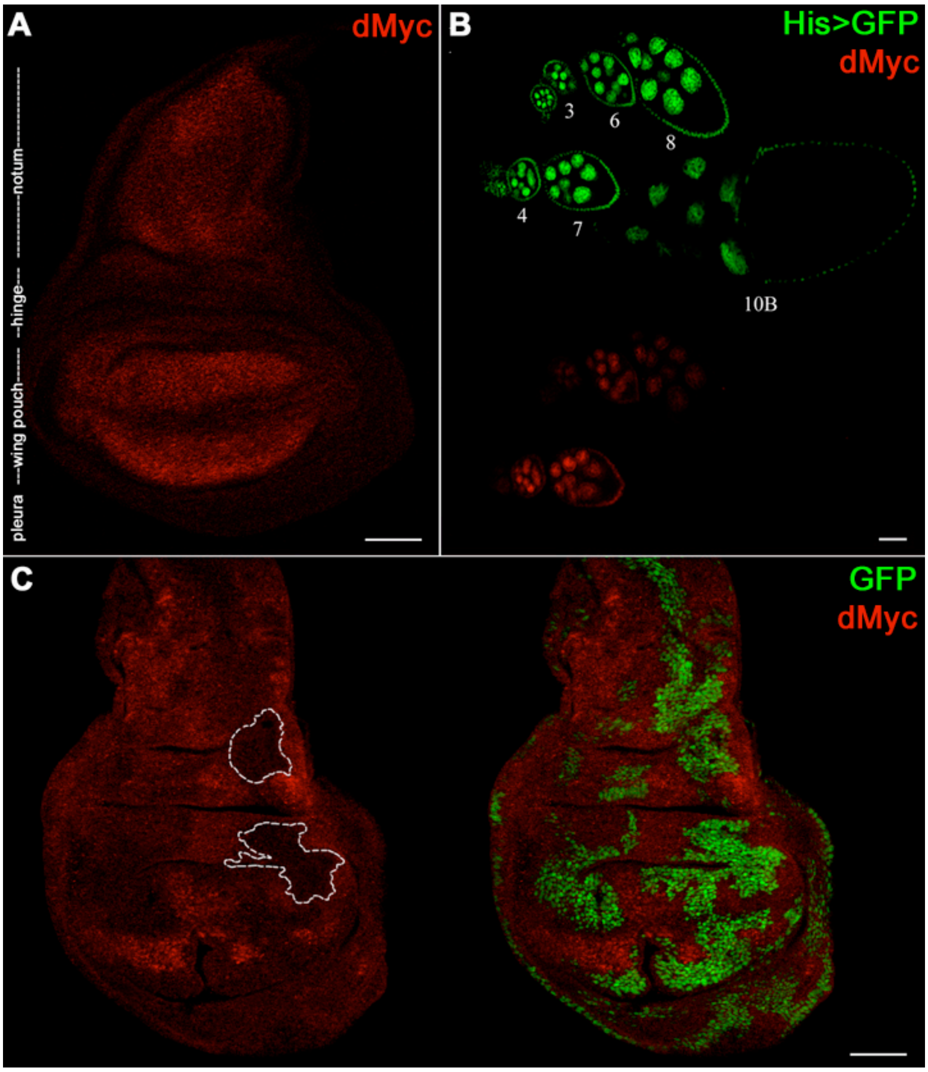

Figure S2. dMyc pattern in wing disc and egg chambers

A: a confocal Z stack of a wild-type, late 3rd instar wing disc stained for dMyc. The presumptive regions of the disc are indicated sideways. B: dMyc protein pattern during oogenesis. Chamber stages are also indicated (King RC: *Ovarian Development in Drosophila melanogaster*. New York: Academic Press; 1970). C: *lgl*$^{-/-}$ clones in *yw*, *hs-Flp*/+, *tub>Gal4*, *UAS-GFP*; *l(2)gl*^{4}, FRT40A/*M(2)24F*, *tub>Gal80*, FRT40A; *UAS-dm*RNAi/+ discs in which the efficacy of the *dmyc* silencing is shown; two clones are outlined as an example. Scale bars are 35 mm.

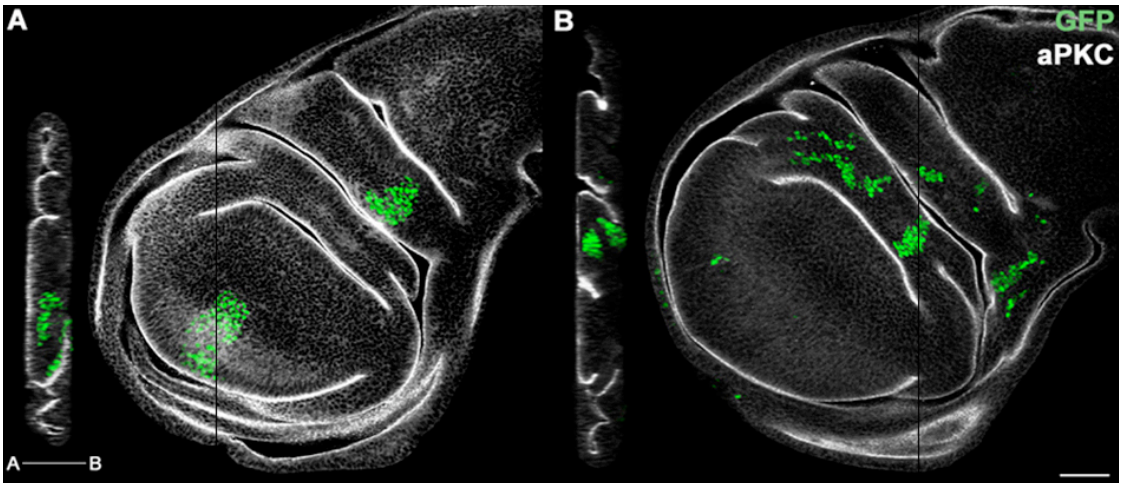

Figure S3. Activated PI3K is not able to rescue the defective growth of *lgl* mutant cells in a wild-type background

A: aPKC staining of *yw*, *hs-Flp*, *tub>Gal4, UAS-GFP*/*UAS-dp110*^{CAAX}; FRT40A/*tub>Gal80*, FRT40A discs. The apical-basal axis of the disc proper is indicated for the Z projection. B: aPKC staining of *yw*, *hs-Flp*, *tub>Gal4, UAS-GFP*/*UAS-dp110*^{CAAX}; *l(2)gl*^{4}, FRT40A/*tub>Gal80*, FRT40A discs in which mutant clones grow poorly leaving the structure of the disc intact. Scale bar is 35 μm.

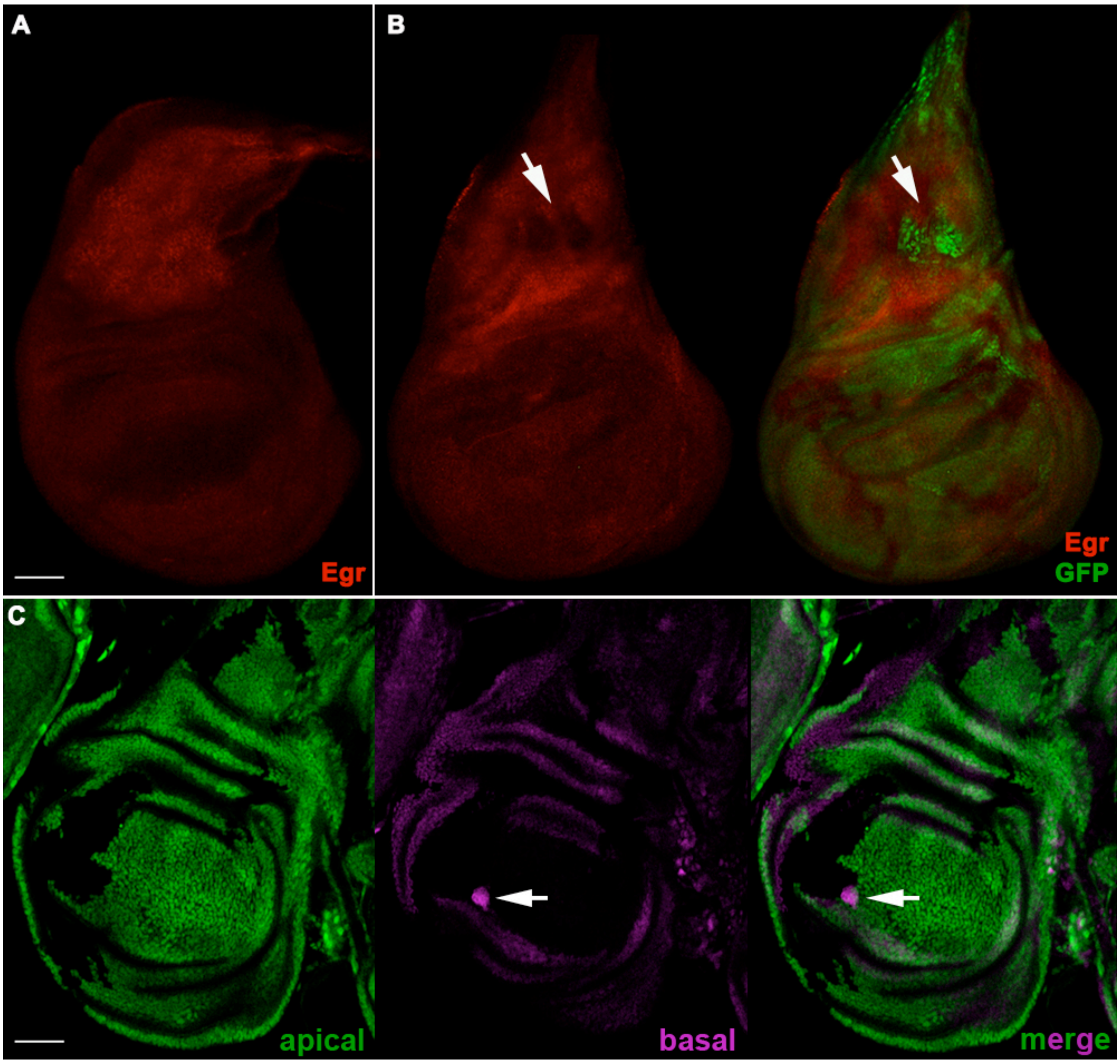

Figure S4. Endocytosis inhibition does not induce *lgl*$^{-/-}$ tumourous overgrowth in the wing pouch region.

In A, Egr pattern is shown in the imaginal wing disc. As previously reported [8], Egr expression is the strongest in the notum region. In B, the arrows indicate *UAS-egr*RNAi mutant tissue (GFP^{2+}) in the notum of a *yw*, *hs-Flp*, *tub>Gal4*/+; *Ubi>GFPnls*, FRT40A /*tub>Gal80*, FRT40A; *UAS-egrRNAi*/+ disc (GFP^{+}). As can be seen, Egr protein is downregulated inside the mutant clone confirming the efficacy of the RNAi construct. In C, the arrows point toward a *lgl*$^{-/-}$; *UAS-YFP::Rab5*^{DN} clone originated in a *yw*, *hs-Flp*, *tub>Gal4*/+; *l(2)gl*^{4}, *Ubi>GFPnls*, FRT40A/*tub>Gal80*, FRT40A; *UAS-YFP::Rab5*^{DN}/+ disc. Apical (green) and basal (false coloured in pink to discriminate the two stacks in the merged figure) sections of the disc show that the mutant clone and its twin are located on different focal planes. Scale bar is 35 μm.

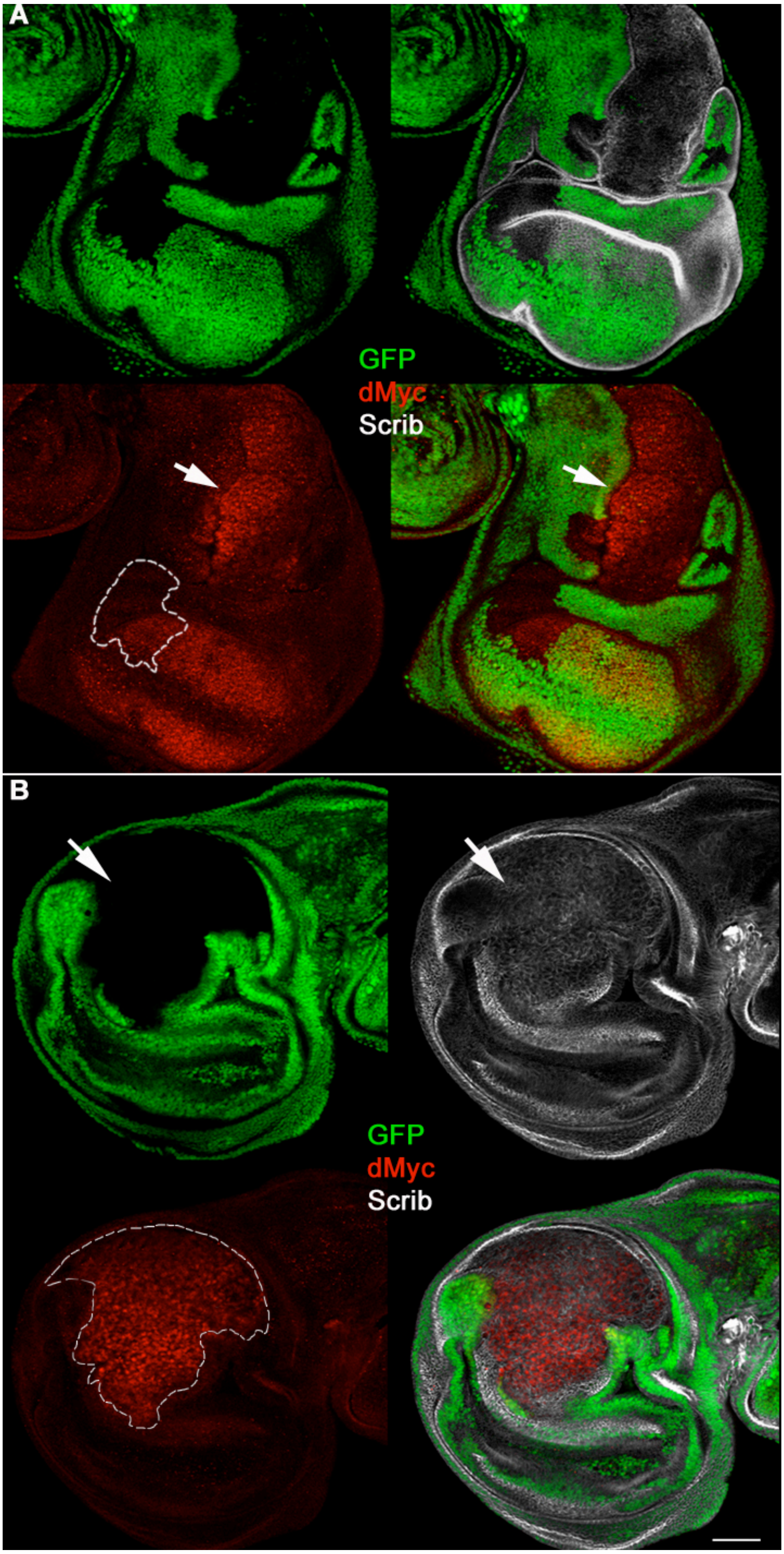
A
GFP
dMyc
Scrib
B
GFP
dMyc
Scrib

Figure S5. *lgl*$^{-/-}$ clonal expansion in a *Minute* background is favoured in those disc regions where dMyc endogenous expression is the lowest

A,B: dMyc and Scrib staining of *w*, *hs-Flp*/+; *M(2)24F*, *Ubi>GFPnls*, FRT40A/*l(2)gl*^{4}, FRT40A wing discs. A: The clone outlined in the wing pouch does not overgrow and shows a dMyc protein level similar to that of the surrounding *M/+, lgl*$^{+/-}$ cells. Clonal expansion is instead visible in the clones originated in the notum (A, arrow) and in the hinge (B) where, as shown by the Scrib staining, cells have lost apico-basal polarity exhibiting a round shape as well as an increase in size (arrow). Clones in the notum and hinge upregulate dMyc protein. Scale bar is 35 µm.

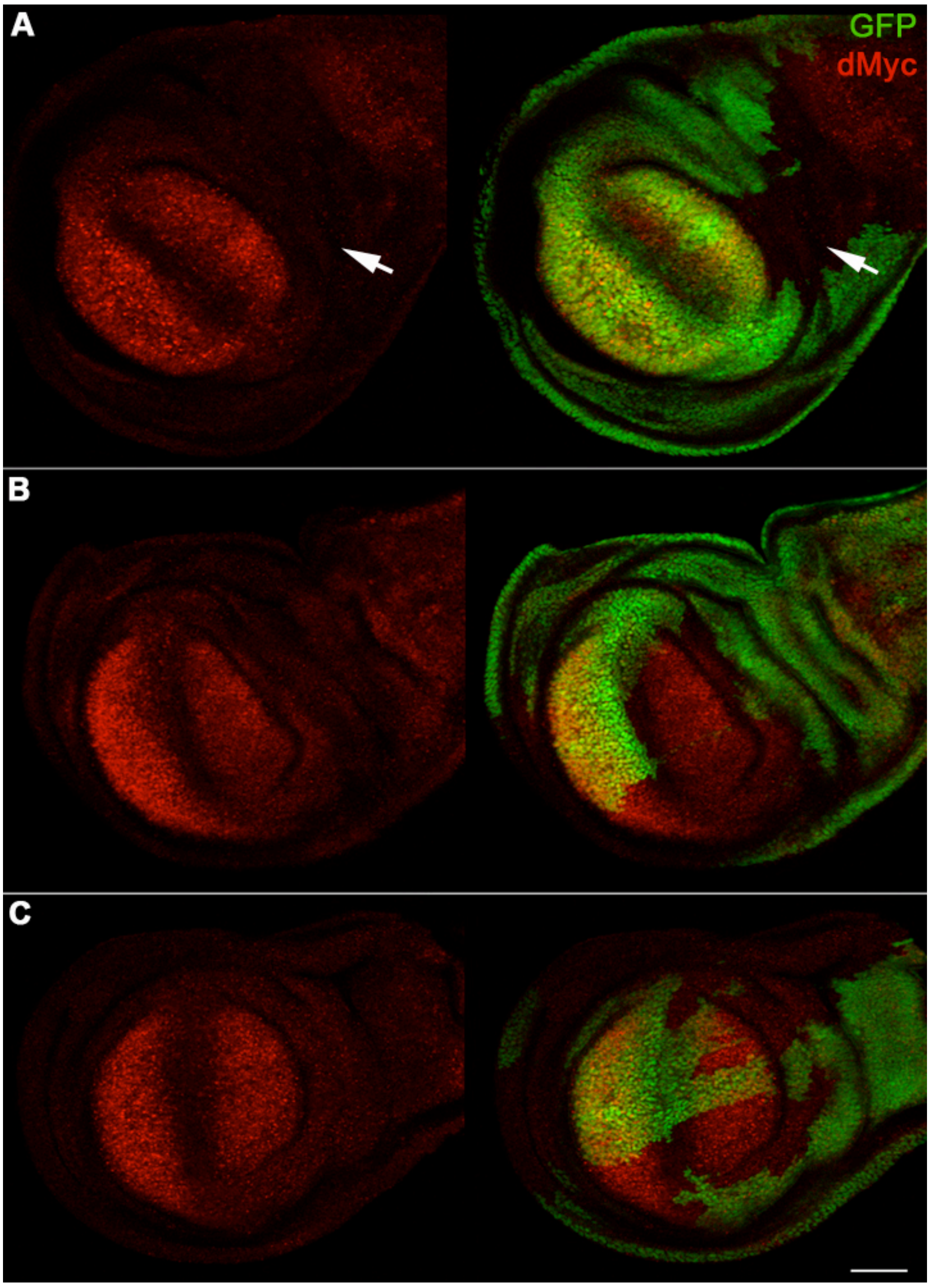

Figure S6. Wild-type and *Minute* cells show comparable levels of dMyc protein in a clonal assay

A-C: dMyc staining of *w*, *hs-Flp*/+; *M(2)24F*, *Ubi*>*GFPnls*, FRT40A/FRT40A discs. No differences in protein level are appreciable in wild-type clones originated in diverse disc regions with respect to the *M*/+ background. The arrow in A indicates a large wild-type clone originated in the hinge showing no dMyc accumulation. Scale bar is 35 μm.

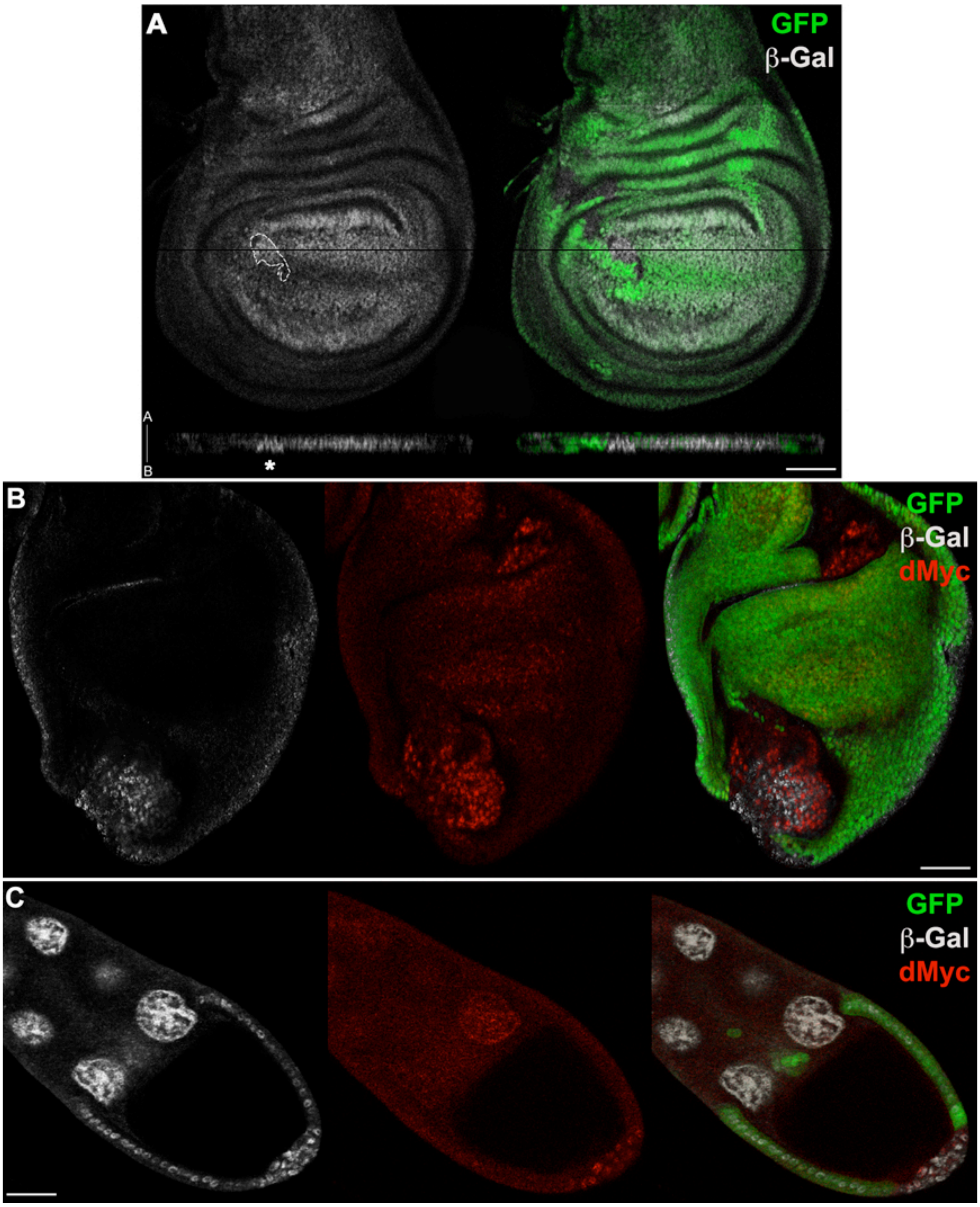

Figure S7. *dmyc* transcriptional activity in *lgl*$^{-/-}$ clones induced in diverse tissues

A: β-Gal staining of *yw*, *dm>LacZ*^{G0354}*/w*, *hs-Flp*; *l(2)gl*^{4}, FRT40A/*Ubi>GFP*, FRT40A discs showing a mutant clone (outlined) in which *dmyc* transcriptional activity does not seem to be impaired. In the projection along the Z axis, in which the apical (A) and basal (B) sides of the disc proper are shown, note that *lgl* mutant nuclei are more basal with respect to neighbours (asterisk). B: β-Gal (white) and dMyc (red) staining of *yw*, *dm>LacZ*^{G0354}*/w*, *hs-Flp*; *l(2)gl*^{4}, FRT40A/*M(2)24F*, *Ubi>GFP*,

FRT40A discs. The *lgl*$^{-/-}$ clone in the pleura shows a *dmyc* transcriptional upregulation in some cells. C: β-Gal (white) and dMyc (red) staining of *yw*, *dm>LacZ*^{G0354}*/w, hs-Flp*; *l(2)gl*^{4}, FRT40A/*Ubi>GFP*, FRT40A egg chambers. The *lgl* mutant clone shows a *dmyc* transcriptional upregulation. Scale bars are 35 μm.

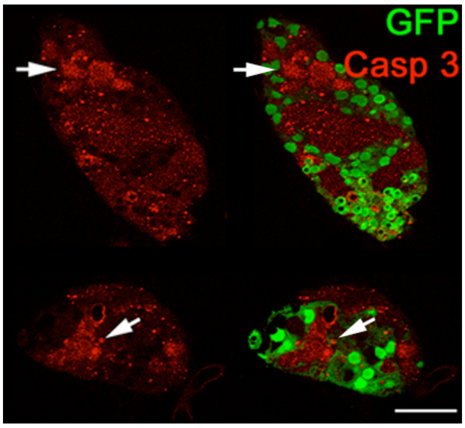

Figure S8. dMyc ectopic expression in ovaries induces cell death in the surrounding tissue

yw, *hs-Flp*/+; *UAS-dmyc*/+; *act>CD2>Gal4*, *UAS-GFP*/+ egg chambers of stage 8 (upper row) and 6 (lower row) in which *dmyc*^{over} Flp-out clones have been induced (GFP^{+}). Arrows indicate groups of wild-type cells included among *dmyc*^{over} clones, in which high levels of active-Caspase 3 staining are visible. No later chambers were recovered due to massive cell death. Scale bar is 35 μm.
